# Congenital Heart Block Maternal Sera Autoantibodies Target an Extracellular Epitope on the α_1G_ T-Type Calcium Channel in Human Fetal Hearts

**DOI:** 10.1371/journal.pone.0072668

**Published:** 2013-09-09

**Authors:** Linn S. Strandberg, Xuezhi Cui, Arianna Rath, Jie Liu, Earl D. Silverman, Xiaoru Liu, Vinayakumar Siragam, Cameron Ackerley, Brenda Bin Su, Jane Yuqing Yan, Marco Capecchi, Luca Biavati, Alice Accorroni, William Yuen, Filippo Quattrone, Kalvin Lung, Edgar T. Jaeggi, Peter H. Backx, Charles M. Deber, Robert M. Hamilton

**Affiliations:** 1 Department of Physiology and Experimental Medicine, The Hospital for Sick Children, Toronto, Ontario, Canada; 2 Division of Molecular Structure and Function, The Hospital for Sick Children, Toronto, Ontario, Canada; 3 Departments of Physiology and Medicine, University of Toronto, Toronto, Ontario, Canada; 4 Scuola Superiore Sant'Anna, Pisa, Italy; 5 Division of Cardiology, University Health Network, Toronto, Ontario, Canada; 6 Department of Biochemistry, University of Toronto, Toronto, Ontario, Canada; Medical University Innsbruck, Austria

## Abstract

**Background:**

Congenital heart block (CHB) is a transplacentally acquired autoimmune disease associated with anti-Ro/SSA and anti-La/SSB maternal autoantibodies and is characterized primarily by atrioventricular (AV) block of the fetal heart. This study aims to investigate whether the T-type calcium channel subunit α_1G_ may be a fetal target of maternal sera autoantibodies in CHB.

**Methodology/Principal Findings:**

We demonstrate differential mRNA expression of the T-type calcium channel *CACNA1G* (α_1G_ gene) in the AV junction of human fetal hearts compared to the apex (18–22.6 weeks gestation). Using human fetal hearts (20–22 wks gestation), our immunoprecipitation (IP), Western blot analysis and immunofluorescence (IF) staining results, taken together, demonstrate accessibility of the α_1G_ epitope on the surfaces of cardiomyocytes as well as reactivity of maternal serum from CHB affected pregnancies to the α_1G_ protein. By ELISA we demonstrated maternal sera reactivity to α_1G_ was significantly higher in CHB maternal sera compared to controls, and reactivity was epitope mapped to a peptide designated as p305 (corresponding to aa305–319 of the extracellular loop linking transmembrane segments S5–S6 in α_1G_ repeat I). Maternal sera from CHB affected pregnancies also reacted more weakly to the homologous region (7/15 amino acids conserved) of the α_1H_ channel. Electrophysiology experiments with single-cell patch-clamp also demonstrated effects of CHB maternal sera on T-type current in mouse sinoatrial node (SAN) cells.

**Conclusions/Significance:**

Taken together, these results indicate that CHB maternal sera antibodies readily target an extracellular epitope of α_1G_ T-type calcium channels in human fetal cardiomyocytes. CHB maternal sera also show reactivity for α_1H_ suggesting that autoantibodies can target multiple fetal targets.

## Introduction

Congenital heart block (CHB) is a passively acquired autoimmune disease that occurs in pregnancies of rheumatic mothers, but also in healthy mothers, and has been associated with maternal anti-Ro/SSA and anti-La/SSB antibodies. The disease is characterized by atrioventricular (AV) block, which can be detected in the developing fetus between 16–25 weeks gestation [1], [2]. In the absence of congenital structural abnormalities in the offspring, maternal autoantibodies are usually present, and it is generally accepted that maternal antibodies cross the placenta and induce fetal injury in the AV node. More generalized effects on the heart, associated with anti-Ro/SSA and anti-La/SSB, have also been suggested in the past decade such as sinus bradycardia, myocardial inflammation, QTc prolongation, endocardial fibroelastosis and dilated cardiomyopathy [3]–[9]. In most studies, untreated autoimmune CHB has been associated with high fetal/neonatal mortality rates (14%–34%) [4], [10]–[16].

Understanding the pathology of CHB, and predicting outcome in pregnancies, have been complicated by low incidence and recurrence rates. In a population of women with anti-Ro/SSA and anti-La/SSB autoantibodies, the incidence of CHB is approximately 1–2% [17], yet the recurrence rate in these mothers is approximately 18% [1], [18], [19], despite persisting antibodies [20], indicating that additional factor(s) contribute to the fetal susceptibility of CHB.

A link between maternal anti-Ro52 antibodies and CHB is supported by a number of studies [20]–[31], whereas maternal sera reactivity to the La protein tends to be associated with dermatologic neonatal lupus erythematosus (NLE) [32]. Nevertheless, both anti-Ro60 and anti-La autoantibodies have been suggested to amplify the immune insult occurring in fetal hearts after development of CHB [33].

CHB pathology reports have shown that the disease is associated with deposition of IgG and with the presence of inflammatory cells in the AV node of fetal hearts, as well as AV node fibrosis and calcification [34]–[36]. Effects on the sinoatrial (SA) node of the fetal heart have also been reported, with sinus bradycardia demonstrated in patients [3], [37], [38], as well as *in vitro* [39] and *in vivo* animal models [40], [41]. Mothers are not affected by AV block, which could be due to developmental expression of the target in the AV node, or unique vulnerability of the fetal heart.

Maternal-fetal antibody transfer as the event precipitating CHB was initially proposed in 1977 [42]. A single protein target for these antibodies, however, has not emerged. Instead, maternal sera reactivity to several proteins has been demonstrated in previous studies, including the serotoninergic 5-HT4 receptor [43], [44], and two voltage-dependent L-type calcium channel subunits: α-1C/Cav1.2 (α_1C_), and α-1D/Cav1.3 (α_1D_) [39], [45], [46]. Sera from mothers with CHB affected pregnancies (CHB^+^ sera) were shown by patch clamp to affect currents mediated by recombinantly expressed α_1C_ in *Xenopus* oocytes [47], and by α_1D_ in a transformed embryonal human kidney cell line (tsA201) [48]. Contribution of L-type channels to CHB was also demonstrated in a mouse model where α_1C_ transgenic and α_1D_ knock out mice were immunized with Ro52, Ro60 and La [49], but these channels did not account for all of the effects of CHB. Results from the *Xenopus* system also indicated that currents through the recombinant voltage-dependent T-type calcium channel subunit α-1H/Cav3.2 (α_1H_) were decreased in the presence of CHB^+^ serum [47]. Since multiple targets of CHB maternal autoantibodies have been identified, and the spectrum of symptoms in affected offspring are not limited to AV block alone, it remains possible that the specific pathogenic autoantibody in anti-Ro/La pregnancies recognizes an epitope that is at least partially common to several ion channels and receptors.

The voltage-dependent T-type calcium channel subunit α-1G/Cav3.1 (α_1G_) is a developmentally regulated channel that is thought to participate along with α-1H/Cav3.1 (α_1H_) regulating cardiac conduction through the AV-node [50], [51]. It is known that α_1G_ is highly expressed in the compact node (CN) of the AV axis in human hearts [52], and homozygous transgenic mice lacking α_1G_ exhibit first-degree AV block and bradycardia [53] – a phenotype consistent with extant reproducible rodent models of CHB [29], [54]–[56].

The suggestion of a role for α_1G_ in cardiac development, and the comparable electrophysiology of the α_1G_ knock out and CHB mouse models, led us to hypothesize that α_1G_ might represent an additional cross-reactive target of maternal serum antibodies in CHB. In the present work, we found that α_1G_ (mRNA and protein) is expressed in human fetal hearts and that CHB-affected maternal sera contains antibodies reacting to the α_1G_ protein. Accessibility of the epitope by serum antibodies is further demonstrated with confocal microscopy and α_1G_ affinity-purified serum. Antibody reactivity of maternal sera maps to peptide sequences found in the extracellular S5–S6 portion of repeat I of α_1G_ (aa305–319), and a similar sequence is found in the α_1H_ T-type channel subunit, but not in the L-type channel subunits α_1C_ or α_1D_. Single-cell patch-clamp studies demonstrated that CHB serum irreversibly decreases T-type calcium channel current in mouse SAN cells. In summary, our results support the conclusion that maternal autoantibodies in CHB pregnancies bind to the T-type calcium channel α_1G_, and can decrease T-type currents. These antibodies may also bind to the α_1H_ channel, but binding to the native protein remains to be verified in human fetal hearts. These results provide further support for the contention that more than one target may be involved in the complex pathology of the disorder.

## Results

### Ion channel blockers demonstrate preferential inhibition of newborn heart AV conduction likely conferred by α_1G_

**Figure 1:**
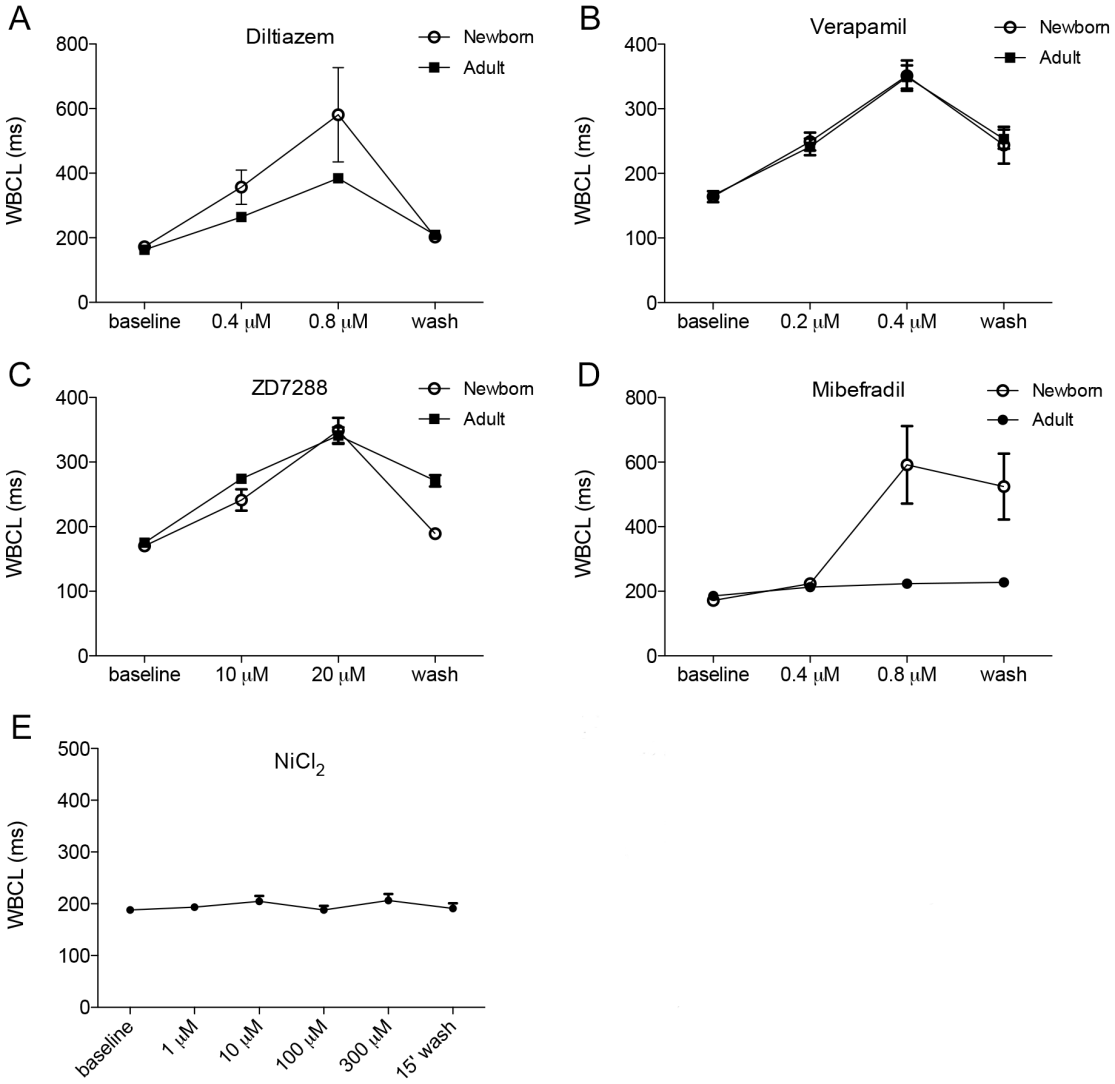
Ion channel blocker effects on Wenckebach cycle length in atrial paced newborn and adult rabbit hearts. Wenckebach cycle length (WBCL) was recorded during perfusion with calcium channel and *I*_f_ channel blockers in newborn and adult rabbit hearts (WBCL in milliseconds). Equivalent effects were observed in adult and newborn hearts treated with L-type calcium channel blockers (**A**) Diltiazem and (**B**) Verapamil and with the *I*_f_ blocker ZD7288 (**C**) (p = *ns* repeated measures ANOVA). The T-type calcium channel blocker Mibefradil (**D**) demonstrated significant preferential AV block in the newborn hearts as compared to the adult hearts (p<0.05). Values shown are mean ± SEM, newborn (n = 7) and adult (n = 8). Diltiazem showed a trend toward a difference in sensitivity of adult and newborn hearts, however there was great variability in results between experiments. (**E**) Effects of the α_1H_ T-type calcium channel blocker NiCl_2_ were also investigated on WBCL in newborn Langendorff rabbit hearts. WBCL measurements do not differ from each other significantly with mean ± SEM (repeated measures ANOVA p = *ns*). One heart out of 6 that appeared to be an outlier showed some variability in response to NiCl_2_, and was excluded from statistical analysis.

Since CHB affects the fetal heart, but not the maternal heart, we wanted to identify ion channels that may be preferentially affected by autoantibodies in fetal hearts. To accomplish this objective we applied selective calcium channel blockers and pacemaker current (*I*_f_) blockers to newborn and adult rabbit Langendorff hearts and measured Wenckebach cycle length (WBCL) by examining the effects of decremental atrial pacing on the P-R interval and AV-conduction block. We have used this method previously to demonstrate that WBCL prolongation occurs with perfusion of sera from mothers of children with CHB, but not in the presence of sera from mothers of unaffected children [57]. We have also used this method previously to establish a greater sensitivity to AV block in newborn versus adult rabbit Langendorff hearts upon perfusion of sera from mothers of children with CHB (RM Hamilton, unpublished observations). Once baseline WBCL was established, hearts were perfused with either L-type calcium channel blockers (Diltiazem or Verapamil), the pacemaker current (*I*_f_) blocker (ZD7288), or, a relatively selective T-type calcium channel blocker (Mibefradil) and 15 minutes after equilibration, WBCL was recorded every five minutes for 15 minutes (Figure 1A–D). The ion channel blocker concentration range was optimized in a pilot study to cause at least double prolongation of WBCL in neonatal rabbit hearts. Baseline WBCLs did not differ significantly in any of the treatment groups (data not shown). Although both newborn and adult hearts are affected by channel blockers, there was no significant difference in WBCL between newborn and adult hearts when they were perfused with L-type calcium channel blockers (Diltiazem, Figure 1A; or Verapamil, Figure 1B), or with the *I*_f_ blocker ZD7288 (Figure 1C). However, newborn hearts were more sensitive to T-type calcium channel blockade (Mibefradil, Figure 1D) compared to adults. Moreover, the percent increase of WBCL in newborn compared to adult hearts during atrial pacing was only increased (p<0.001) with Mibefradil. In order to evaluate whether Mibefradil-induced AV Wenckebach block resulted from blockade of α_1G_ or α_1H_, we also measured WBCL during perfusion with increasing concentrations of nickel chloride (NiCl_2_), because α_1G_ and α_1H_ have different sensitivities to Ni^2+^ block (IC_50_ = 13 µM for α_1H_ and IC_50_ = 250 µM for α_1G_) [58]. Since WBCL was unaffected by Ni^+^ up to concentrations of 300 µM (Figure 1E) in 5/6 hearts, we conclude that α_1G_ is primarily responsible for the Mibefradil effects on WBCL prolongation in newborn vs. adult hearts.

### α_1G_ expression in the AV node of human fetal hearts

**Figure 2:**
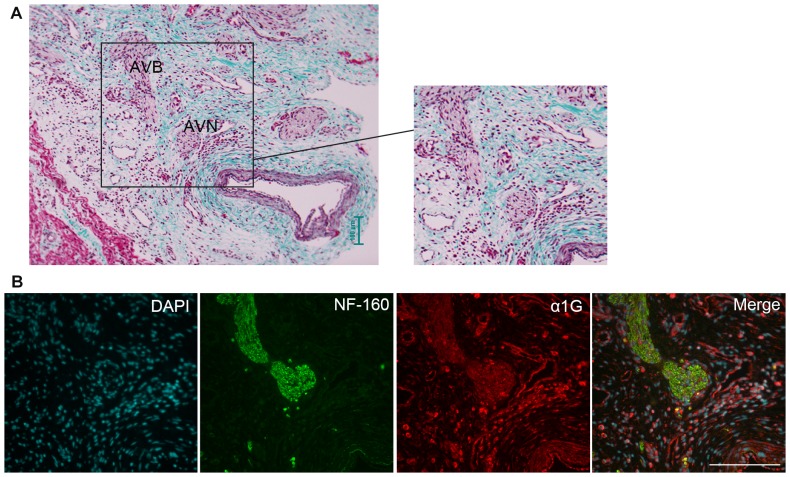
T-type calcium channel α_1G_ is expressed in the AV node of human fetal hearts. (**A**) Masson's trichrome staining of 21-week human fetal heart demonstrates morphology of the AVJ region with light pink staining of the spindle-like cells in the AV node (AVN) and the AV bundle (AVB), green staining corresponding to collagen fibres. (**B**) α_1G_ staining (Red) is present in AVJ and AV bundle regions which are distinguishable by positive NF-160 staining (Green). Scale bars represent 100 µm (A) and 150 µm (B).

Since the expression of α_1G_ in the AV node of the human fetal heart is a prerequisite for any potential involvement of this protein in the AV block characteristic of CHB, we performed anatomical dissection, as previously described [59], and immunohistochemistry (IHC) studies to demonstrate presence of α_1G_ in the AV node. Sections from a 21-week human fetal heart with Masson's trichrome stain demonstrates morphology of the AVJ region, clearly showing the AV node and AV bundle, which coincides with appropriate anatomical regions identified by collagen (green) and myocardium (light pink) (Figure 2A). The NF-160 antibody (green), which identifies cardiac conducting tissue [60], clearly shows the AV node and AV bundle (Figure 2B). We observed dense staining with α_1G_ antibodies (red) in the region of the AV node and AV bundle with more diffuse staining in surrounding regions corresponding to ventricular myocardium identified as regions without NF-160 staining (Figure 2B). Due to lack of suitable antibodies, we were not able to perform IHC to investigate expression in the AV node of the other T-type channel subunit expressed in the heart, α_1H_.

### α_1G_ gene expression is enriched vs. α_1H_ in the atrioventricular junction

**Figure 3:**
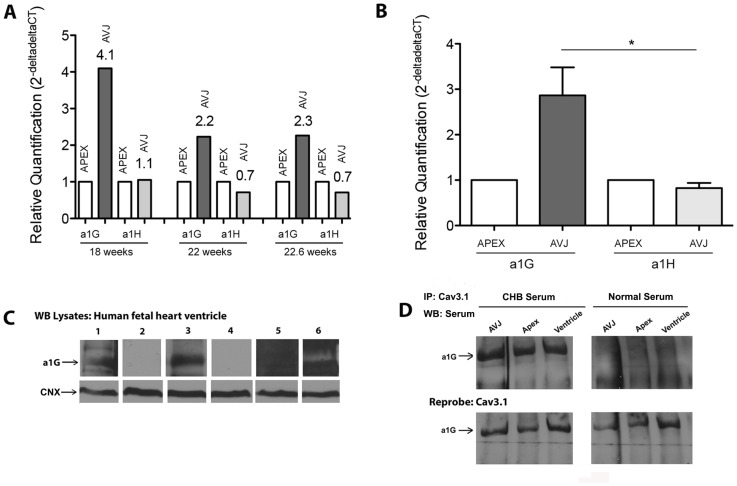
Expression analysis, and CHB maternal sera immune reactivity towards α_1G_. (**A**) Although both α_1G_ and α_1H_ are expressed in human fetal hearts, real time PCR demonstrates that transcripts from *CACNA1G* (α_1G_) are 2.2- to 4-fold higher in the AVJ than in the apex tissue (between 18–22.6 weeks gestation), whereas *CACNA1H* expression levels are between 0.7- and 1.1-fold in the AVJ compared to the apex. (**B**) Combining the data from 3 hearts (18.0 weeks-22.6 weeks), shows that *CACNA1G* expression in the AVJ is significantly higher than *CACNA1H* in the AVJ (p<0.05). (**C**) Western blot with human fetal heart lysate (20.4 weeks) demonstrates α_1G_ expression by a Cav3.1 commercial antibody (lane 1), that is blocked by the peptide immunogen of this antibody (aa1–22 of rat α_1G_) (lane 2). CHB sera (anti-Ro and anti-La titer >100 IU) also binds to α_1G_ (lane 3); this reactivity is blocked by the α_1G_ p305 peptide (lane 4). Sera from mothers with anti-Ro/La antibodies (anti-Ro = 60 IU and anti-La titer >100 IU) giving birth to normal babies do not have immune reactivity to the α_1G_ protein (lane 5), and a commercial α_1H_ antibody confirms that the band seen is not α_1H_. (**D**) α_1G_ isolated from human fetal heart (AV junction, ventricle, and apex) was immunoprecipitated with a Cav3.1 antibody, and the immunoblot was probed with human sera. Immunoblot was subsequently stripped and re-probed with a second Cav3.1 antibody towards a different epitope, demonstrating presence and specificity of the IP towards α_1G_. Note that CHB sera is defined as anti-Ro^/^La positive sera from pregnancies affected by CHB. Normal sera are from pregnancies with a healthy outcome and not affected by CHB.

To further explore the expression of α_1G_ channels in fetal human hearts, we anatomically dissected [59] atrioventricular junction (AVJ) and the ventricular apex from three fetuses between 18–22.6 weeks gestation (18 weeks, 18.6 weeks, 22.6 weeks), coinciding with the time point when CHB is diagnosed *in utero*. Real time PCR measurements revealed that the expression of the α_1G_ genes (*CACNA1G*), normalized to the level of expression in the apex, is higher at 18 weeks compared to later time points (Figure 3A). Although the availability of only a single sample per gestational week does not permit us to assess the reproducibility of these expression patterns, pooling of the results supports the conclusion that the relative expression of *CACNA1G* is greater (p<0.05) in the AVJ compared to the ventricular apex (Figure 3B). For comparison, we also measured the mRNA expression levels of the α_1H_ gene, *CACNA1H*, and could not detect higher expression in the AVJ versus the ventricular apex, suggesting low expression of α_1H_ in the AVJ.

### Maternal sera autoantibodies from CHB-affected pregnancies are immunoreactive to the α_1G_ protein in human fetal hearts

In order to investigate reactivity of CHB maternal sera to α_1G_, we performed Western blot and immunoprecipitation experiments with lysates from fetal heart ventricle. We first confirmed presence of α_1G_ protein in human fetal hearts by Western blot with lysates from a 20.4-week-old human fetal heart. A commercial α_1G_/Cav3.1 antibody (specific for aa1–22 of α_1G_) identified a band ∼180–200 kDa in size (Figure 3C, lane 1) which was eliminated by pre-incubation with a peptide corresponding to aa1–22 of rat α_1G_. Blotting with anti-Ro/La positive CHB^+^ maternal sera (titer >100 IU for both anti-Ro and anti-La) also identified a similar band (∼180–200 kDa, Figure 3C, lane 3) that was eliminated by pre-incubation with peptide p305 (aa305–319 of α_1G_, see below). Serum obtained from a mother positive for anti-Ro/La autoantibodies (anti-Ro titer = 60 IU and anti-La titer >100 IU), but with an unaffected fetus, did not yield a band with the expected molecular weight of α_1G_ (Figure 3C, lane 5, no bands). We also show in Figure 3C (lane 6) that a commercial antibody to α_1H_ revealed a band, but the predicted molecular weight was much smaller than α_1G_ (180–200 kDa). Note further that proper loading in all the wells was ensured using control anti-calnexin (CNX) antibody (Figure 3C, bottom panel).

The presence of α_1G_ in human fetal heart lysates, and verification of immune reactivity by CHB maternal sera, was next assessed via immunoprecipitation (IP) experiments. IP was performed with the commercial α_1G_/Cav3.1 antibody (specific for aa1–22 of α_1G_) on lysates prepared from three dissected regions of human fetal hearts (AVJ, ventricle, apex). Immunoblots of IP samples from the AVJ, ventricle and apex, yielded α_1G_ bands when probed with commercial α_1G_/Cav3.1 antibody and maternal serum from an anti-Ro/La positive pregnancy with CHB outcome (CHB serum, titer >100 IU for both anti-Ro/La), but not with maternal serum from an anti-Ro/La negative healthy pregnancy (Normal serum) (Figure 3D). These blots were subsequently stripped and re-probed with a second commercial α_1G_/Cav3.1 antibody recognizing a different α_1G_ epitope (specific for aa6–50), demonstrating specificity of the α_1G_ IP. We were unable to find appropriate conditions, or antibodies, that yielded evidence for the presence of α_1H_ protein levels with IP; and, although Western blot did demonstrate a band for α_1H_, the size of this band differed from that recognized by CHB^+^ maternal sera.

### Accessibility of α_1G_ T-type calcium channel epitope on cardiomyocytes

**Figure 4:**
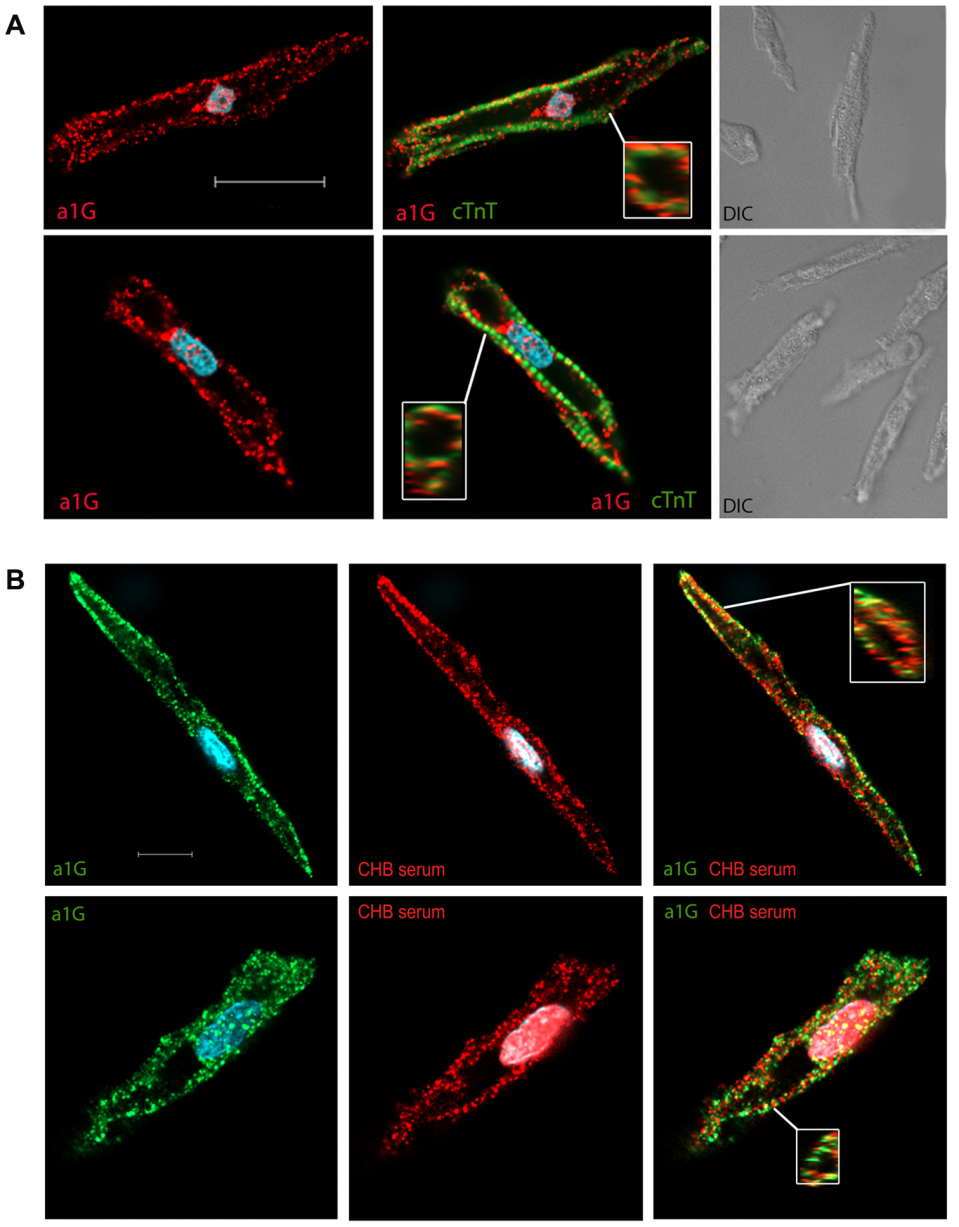
Affinity-purified maternal CHB serum antibodies co-localize with α_1G_. (**A**) Cardiomyocytes from ventricle of human fetal hearts, gestational week 20.6, were dissociated, immunofluorescence stained, and visualized with confocal microscopy. Cardiomyocytes are stained for Cav3.1 (α_1G_) (Red), for the cardiomyocyte marker anti-Troponin-T (Green), and nuclei visualized with DAPI (Blue). Secondary antibodies alone gave no stain. Inset image demonstrates expression on the surface in a cross section image. Images shown are representative of various cardiomyocyte samples; similar staining patterns were obtained in four replicate experiments. Secondary antibodies, anti-rabbit-IgG-Cy3 (Red) and anti-mouse-IgG-Alexa488 (green) alone give no stain (data not shown). Note that DAPI stains the nuclei of cardiomyocytes and of other cells present in the samples, such as fibroblasts. Scale bar represents 12 µm. (**B**) Dissociated and non-permeabilized cardiomyocytes from ventricle of 20.6 week human fetal hearts, immunofluoresence stained for α_1G_ affinity-purified serum (red), and α_1G_ (green). Co-localization of staining (yellow) was assessed with confocal microscopy. Scale bar represents 12 µm.

Because our RT-PCR and Western Blotting studies showed convincingly the expression of α_1G_ in the ventricles, we elected to explore whether α_1G_ affinity-purified CHB sera identified surface targets in the myocardium using immunofluorescence and confocal microscopy. Although CHB is primarily characterized by atrioventricular block affecting the AV node, effects of anti-Ro/La autoantibodies have also been suggested on the whole heart [3]–[9], [61], thus expression of targets in regions such as the ventricle are also of interest. Immunofluorescence co-staining of human fetal heart (21.6 weeks) with anti-α_1G_ (Red) and with anti-cTnT antibodies (Green) exhibited preferential staining of α_1G_ localized predominantly to the cell surface, with some perinuclear staining observed by confocal microscopy (Figure 4A). Differential intereference contrast (DIC) microscopy images of cardiomyocytes used for staining experiments demonstrate typical morphology of ventricular cardiomyocytes (Figure 4A, right panel). Maternal CHB sera (titer >100 IU for both anti-Ro and anti-La), that had been affinity-purified towards the α_1G_ peptide p305 (aa305–319, see below), preferentially stained surface regions of the ventricular cardiomyocytes (yellow, Figure 4B, right panel). Interestingly, some surface regions only showed α_1G_ staining with the commercial antibody, and not co-staining with the affinity-purified sera, suggesting that the α_1G_ epitope recognized by the maternal sera may be masked (or even absent) in some membrane areas.

### CHB maternal sera profile screening of α_1G_ peptides

In order to better understand the patterns of antibody reactivity to α_1G_ described above, we undertook to delineate the α_1G_ epitope(s) recognized by CHB maternal sera. The α_1G_ protein is comprised of 24 transmembrane segments organized into four homologous domains (termed repeats I–IV); consequently, the protein has many extracellular (12) and intracellular sequences (13) [62]. The first 4 transmembrane segments of each internal repeat (S1–S4) form the voltage-sensors, whereas the S5–S6, and the intervening extracellular loop, form the pore [62].

**Figure 5:**
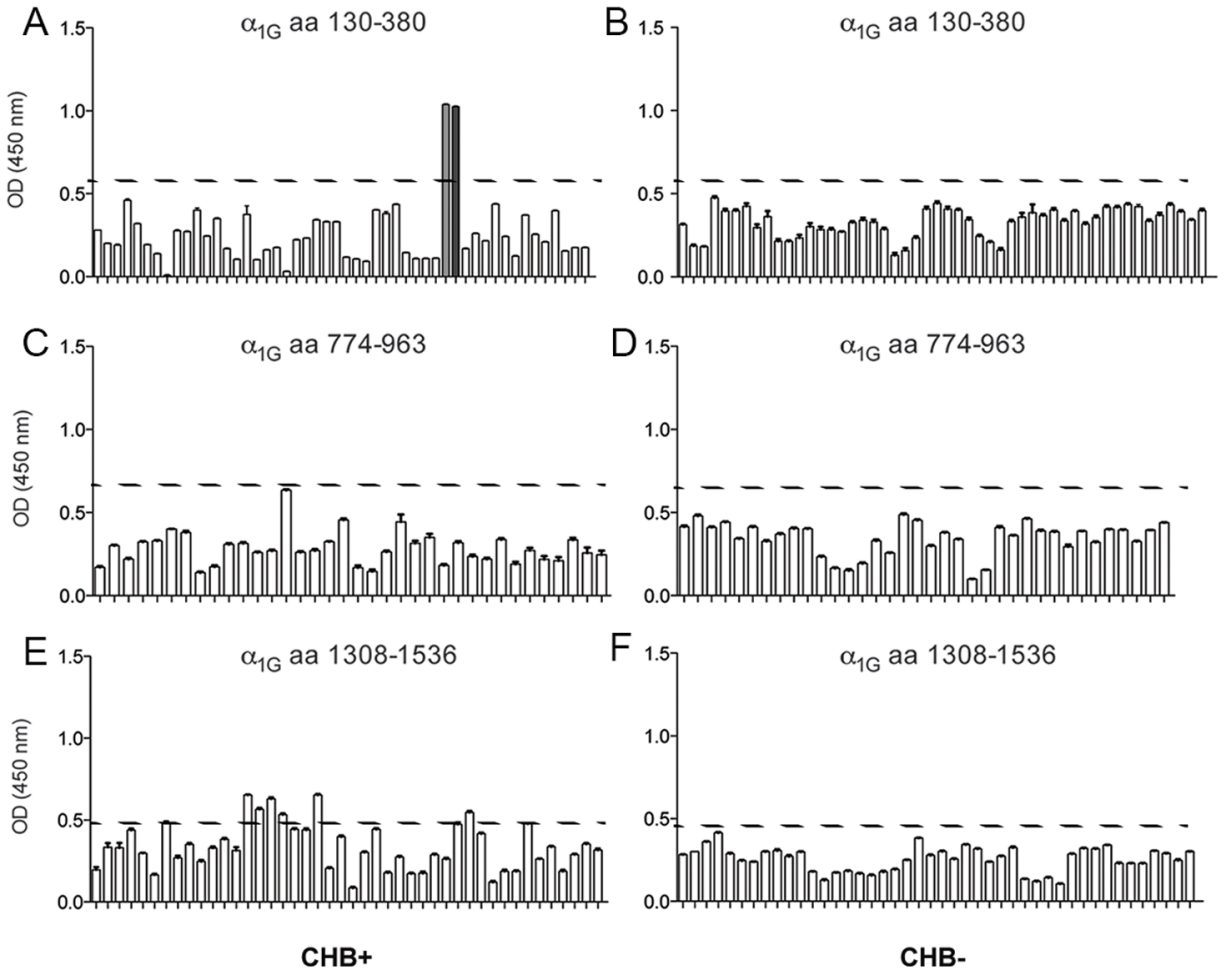
Maternal sera antibody profile screening of α_1G_ peptides. Maternal sera from one CHB^+^ pregnancy (**A, C, E**) and one CHB^−^ pregnancy (**B, D, F**) were tested in ELISA against 15aa long overlapping peptides derived from extracellular regions of α_1G_: T-type aa130–380 (**A, B**); aa774–963 (**C, D**); and, aa1308–1536 (**E, F**). Reactivity above threshold (HC: Average+3×St. Dev; set at 0.59, 0.64, 0.48 respectively for A/B, C/D, and E/F) was observed in the CHB^+^ but not CHB^−^ serum to α_1G_ peptides p305 (aa305–319, light grey bar) and p315 (aa315–323, dark grey bar) was observed in the CHB^+^ but not the CHB^−^ serum. Above threshold reactivity was also observed among peptides in the region spanning aa1308–1536.

Given that CHB-derived antibodies stained surface-expressed α_1G_ in cardiomyocytes, it seemed reasonable to conclude that this sera recognizes extracellular epitopes of the α_1G_ protein. Accordingly, a PEPscreen (Sigma) custom peptide library consisting of 15aa-long overlapping peptides was generated that encompassed selected regions of the α_1G_ protein [aa130–380 (Figure 5A, B)]; II [aa774–963 (Figure 5C, D)]; and, III [aa1308–1536 (Figure 5E, F)] corresponding to the largest extracellular loops formed between S5 and S6 of repeat domains I, II and III. Sera from three mothers with CHB affected pregnancies, and three patients with pregnancies unaffected by CHB (denoted CHB^+^ and CHB^−^, respectively), were subsequently screened for reactivity to the peptide library (see Figure 5 for representative data). We identified two peptides in repeat I, zero peptides in repeat II, and six peptides in repeat III with levels of reactivity that exceeded background (Figure 5, A–C); no reactivity was observed with CHB^−^ sera (Figure 5, D–F). The magnitude of response of CHB^+^ sera differed between peptides based on repeat I and repeat III. For example, all the peptides derived from repeat I exceeded background by ∼80–100%, whereas the most reactive repeat III peptide read out was ∼20% above background (compare Figure 5A and Figure 5C). We therefore chose to continue our epitope mapping to the region of α_1G_ repeat I encompassed by the two highly reactive peptides. These peptides, denoted p305 (aa305–319, Figure 5A, light grey bar) and p315 (aa315–325, Figure 5A, dark grey bar), are comprised of sequences predicted to reside between S5 and S6 in the third extracellular loop of repeat I.

**Figure 6:**
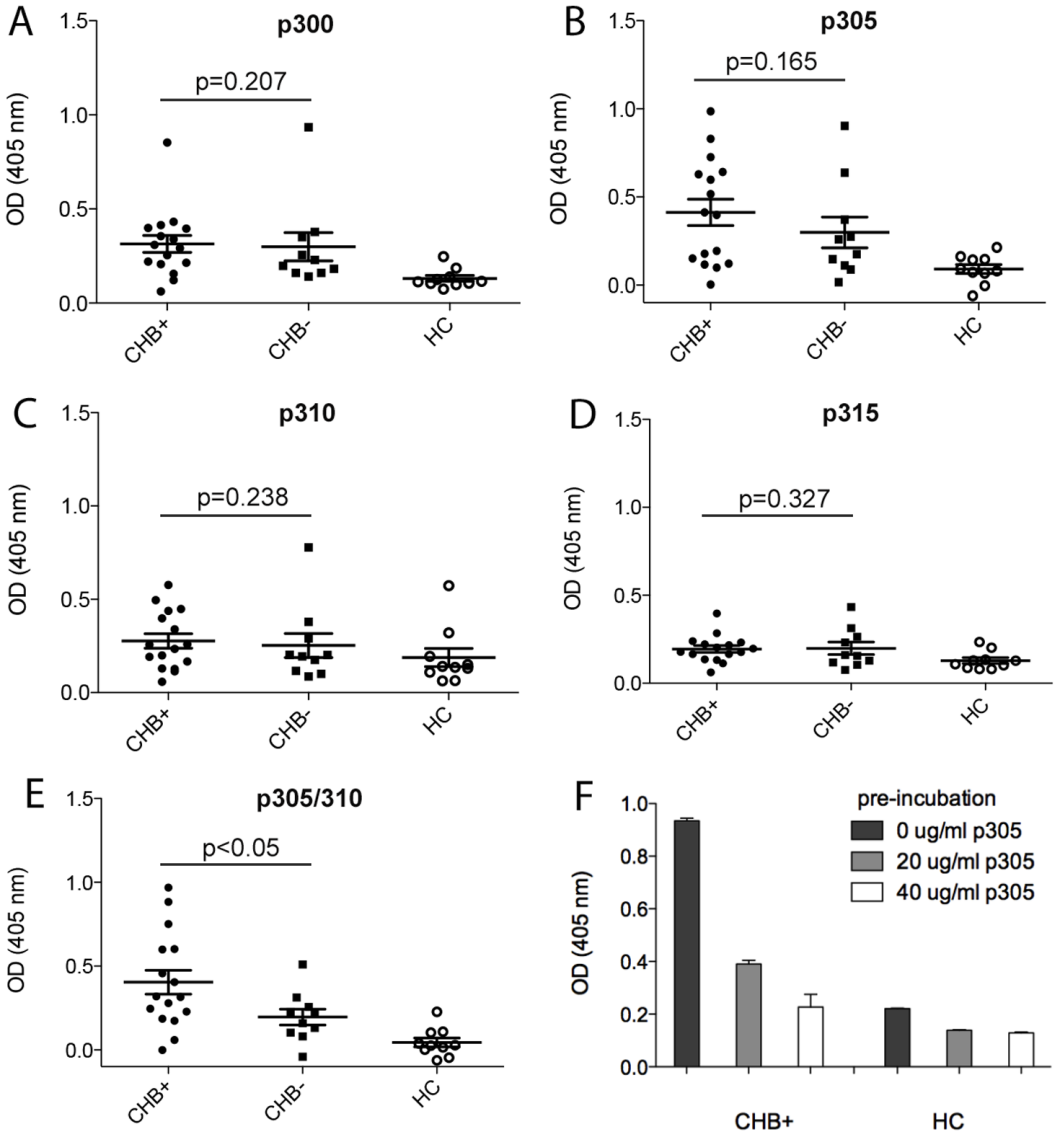
Reactivity in CHB pregnancies is specific for the p305 peptide of α_1G_ and can be blocked. Four overlapping 15aa peptides, and one 20aa peptide were selected to further characterize maternal sera reactivity to α_1G_ in mothers with CHB pregnancies (CHB^+^) compared to mothers with unaffected pregnancies (CHB^−^). Reactivity to (**A**) peptide p300 (aa300–314), (**B**) p305 (aa305–319), (**C**) p310 (aa310–324), (**D**) p315 (aa315–329) demonstrate that sera from mothers with pregnancies affected by CHB (CHB^+^), have significantly higher p305 antibody levels compared to unaffected (CHB^−^) pregnancies (p<0.05). Although p305 (aa305–319) had the highest reactivity, a longer peptide combining p305 and p310 designated p305/310 (aa305–24) demonstrated a significant difference between CHB^+^ and CHB^−^ maternal serum (**E**). Pre-incubation of one CHB^+^ maternal sera, and one healthy control (HC) sera with increasing concentration (0, 20, 40 µg/ml) of peptide p305 (aa305–319) demonstrates that the reactivity of sera is specific for this peptide and can be blocked (**F**). Error bars indicate mean ± SE.

Epitope mapping of the repeat I peptides was refined in enzyme-linked immunosorbent assay (ELISA) experiments that utilized four overlapping 15aa peptides, and one 20aa peptide, with sequences corresponding to this protein region, as follows: p300 (aa300–314); p305 (aa305–319); p310 (aa310–324); p315 (aa315–329); and, p305/310 peptide (aa305–324). Assay parameters were developed using healthy control sera as a negative control, with the positive control provided by serum from a patient clinically identified as anti-Ro/La positive and confirmed as immunoreactive to α_1G_ by immunoblotting (Figure 3C, lane 1). Using these controls, a reproducible ELISA screen for each peptide was established, and the reactivity of small cohorts of maternal CHB^+^ and CHB^−^ serum samples to each peptide was investigated (Figure 6A–E).

Maternal sera reactivity from CHB^+^ or CHB^−^ pregnancies was not different towards peptides p300 and p315 (Figure 6A and Figure 6D, respectively), which were also the slowest-reacting peptides (read at 2 h 30 min). However, CHB^+^ sera were far more reactive to p305, p310, and p305/310 than CHB^−^ sera (Figure 6B, C, E) with the greatest reactivity (Figure 6E, p<0.05) seen for the p305/310 peptide. Moreover, pre-incubation of the CHB^+^ maternal sera with p305 peptide reduced reactivity on ELISA, indicating specificity (Figure 6F). These results support the conclusion that p305 and p305/310 contained the predominant epitope(s) of α_1G_ recognized by CHB^+^ maternal sera.

### Protein structure and identity in S5–S6 extracellular loop regions in T- and L-type calcium channels

**Figure 7:**
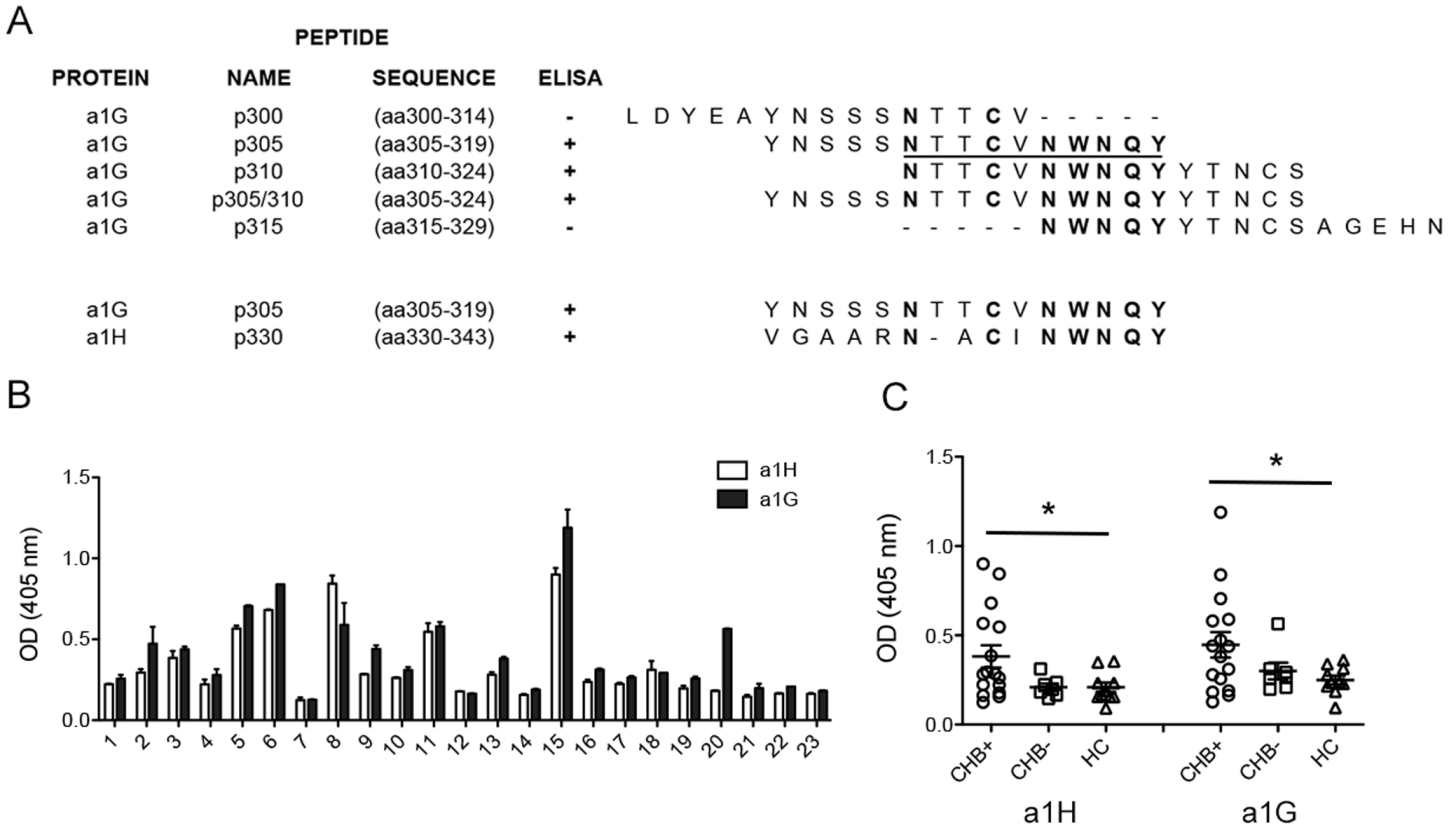
Sequence identity and maternal sera reactivity for α_1G_ and α_1H_ T-type calcium channel peptides. (**A**) Alignment of peptides derived from human α_1G_ and α_1H_ S5–S6 extracellular loop I sequences. The ‘core sequence’ of interest with 10 amino acids in common among the α_1G_ peptides is underlined, and the seven residues that are identical in the α_1G_ and α_1H_ sequences are indicated in bold type. Note that this group is present in the sequence of each immunoreactive peptide, whereas the non-reactive peptides lack two or more of these amino acid residues. Sequences shown correspond to human CAC1G (α_1G_) and human CAC1H (α_1H_) (UniProt accession numbers O43497 and 095180, respectively). (**B**) Peptides derived from the aligned loop regions of α_1G_ and α_1H_ exhibit a similar pattern of CHB^+^/CHB^−^ reactivity. CHB maternal sera ELISA reactivity (OD 405 nm) was plotted with interleaved bars for comparison. Error bars represent Mean ± SE. (**C**) Reactivity of CHB^+^ maternal sera was observed to be significantly higher than HC sera, where p<0.05 for both α_1G_ p305 (aa305–319) and α_1H_ p330 (aa330–343), but did not reach significance for comparison with CHB^−^ sera (p = 0.075 and p = 0.184 for α_1G_ and α_1H_, respectively).

Inspection of the patterns of the CHB^+^ maternal sera reactivity to the peptides revealed that the sequence NTTCVNWNQY, herein referred to as the ‘core sequence’, was common to all the reactive peptides (Figure 7A). An alignment of the α_1G_ and α_1H_ proteins revealed that α_1H_ contains a similar peptide sequence in the S5–S6 loop in repeat I with sequence identity for NWNQY, as well as nearby amino acids, with the conserved Cys residue being implicated previously in channel gating activation, inactivation, and deactivation [63].

By contrast, alignments of α_1G_ with the L-type calcium channel α_1C_ and α_1D_ sequences did not uncover evidence of sequence similarity (data not shown). It is important to note that examination of the peptide sequences of the Ro52, Ro60 and La autoantigens, despite their association with CHB, do not have comparable sequences in α_1G_, α_1H_, α_1C_ or α_1D_. However, only 2% of Ro/La positive pregnancies are affected by CHB, supporting the conclusion that these antibodies are not specific to the CHB outcome alone.

**Figure 8:**
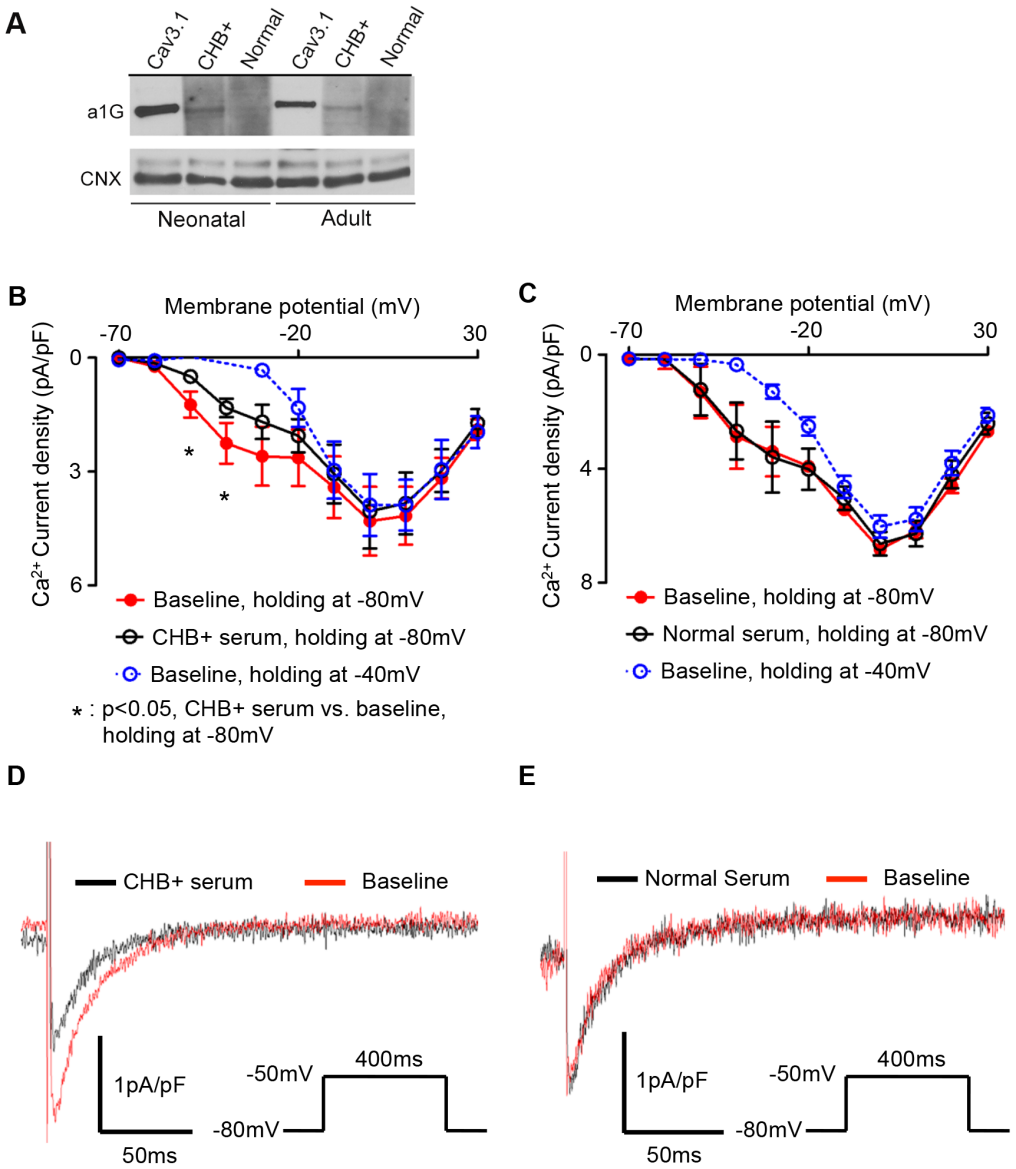
Effect of maternal sera on calcium current in mouse sinoatrial node (SAN) cells. (**A**) CHB maternal serum antibodies, but not healthy control serum, show reactivity to mouse α_1G_ in Western blot. Commercial α_1G_ (Cav3.1) antibody demonstrates the α_1G_ positive band and calnexin (CNX) antibody probing shows equal loading of mouse heart lysate. (**B**) Calcium current density-voltage curve with CHB serum, (**C**) Calcium current density-voltage curve with normal serum, (**D**) representative Calcium current traces recording at −50 mV from holding at −80 mV with CHB serum, (**E**) representative Calcium current traces recording at −50 mV from holding at −80 mV with normal serum. CHB serum is defined as an anti-Ro^/^La positive serum from a pregnancy affected by third-degree AVB or CHB. Normal serum was obtained from a mother with a pregnancy unaffected by CHB, resulting in a healthy outcome.

To evaluate whether the core sequence has the potential to form a protruding epitope for antibody binding, we employed the ElliPro Epitope Modeling Analysis Tool (http://tools.immuneepitope.org) which predicts linear and discontinuous antibody epitopes based on a protein antigen's 3D structure [64]. We attempted to avoid bias towards the core sequence by submitting the entire sequence of the S5–S6 extracellular loop of α_1G_ repeat I (aa235–370, UniProt) to this server for epitope prediction. Six potential linear epitopes were identified; one of these had the sequence SSSNTTCVNWNQYY, which obviously encompasses the core sequence, and is nearly identical in sequence to p305 (see Figure 8A). We also submitted the same region to the Kolaskar & Tongaonker Antigenicity prediction and found that the eight amino acids NTTCVNWN, present in our “core sequence”, were predicted to be an antigenic peptide based on a method predicting antigenicity from the expression of certain hydrophobic amino acids present on the surface of a molecule [65].

### CHB^+^ Maternal sera are immunoreactive to peptides derived from the S5–S6 extracellular loops of α_1G_ and α_1H_ repeat I

Based on the results above, we hypothesized that CHB^+^ maternal sera might react with the S5–S6 segment in repeat I of α_1H_. A peptide corresponding to the aligned region of α_1H_ (p330, aa330–343, Figure 7B) was accordingly utilized to screen a group of 23 CHB^+^ maternal sera, and the reactivity profile compared to that of p305. The pattern of reactivity obtained with each peptide was qualitatively similar (Figure 7B), and the levels of α_1H_ reactivity observed among the sera were significantly correlated to those of α_1G_ (Spearman correlation, r = 0.89, p = 0.0001). The parallel reactivity of CHB^+^ sera towards p305 and p330 led us to hypothesize that p330 might also provide a means to distinguish between CHB^+^ and CHB^−^ sera. Screening of maternal sera with p305 (α_1G_) and with p330 (α_1H_) indicated a tendency for the CHB^+^ and CHB^−^ groups to have differential reactivity for the peptides, although these results were of marginal significance (p = 0.075 and 0.184 for p305/α_1G_ and p330/α_1H_, respectively). Each peptide was nevertheless capable of distinguishing between the CHB^+^ and healthy control (HC) groups (p<0.05 for both peptides, Figure 7C). These results support the conclusion that CHB^+^ sera can also react with α_1H_, as well as α_1G_, consistent with previous studies [66]. This conclusion is consistent with our RT-PCR and Western blot studies (Figure 3) establishing expression of α_1H_ in human fetal hearts.

### Electrophysiological recordings of T-type current in SAN cells

To evaluate a functional effect of CHB^+^ maternal serum on T-type Ca^2+^ current, we isolated sinoatrial node (SAN) cells from mice. Single-cell patch-clamp experiments were performed on adult mouse heart SAN cells to isolate Ca^2+^ currents since protein analysis demonstrated expression of α_1G_ in both neonatal and adult mouse (Figure 8A). As expected from previous reports [67], [68], the Ca^2+^ current began activating at voltages negative to −50 mV with the current-voltage relationship showing clear evidence for the presence of two components when holding at −80 mV, which became one component at holding potentials of −40 mV. Previous studies have established that the first component activated at the more negative potentials (between −60 mV and −40 mV) is a signature of T-type calcium currents in SAN pacemaker myocytes [69]. Importantly, the application of CHB^+^ serum irreversibly and preferentially decreased (p = 0.0313) Ca^2+^ current at voltages between −60 and −20 mV, precisely where T-type calcium currents are differentially activated (Figure 8B, D). At −50 mV, the CHB^+^ serum reduced the Ca^2+^ current from −1.23±0.34 pA/pF to −0.48±0.15 pA/pF (n = 6) while no significant change (p = 0.8772) was observed when normal serum was added (baseline: −1.32±0.90 pA/pF, control serum: −1.22±0.81 pA/pF; (Figure 8C, E; n = 6). CHB^+^ serum also decreased (p = 0.0438) current at −40 mV from −2.25±0.53 pA/pF to −1.32±0.24 pA/pF (n = 6). At more positive potentials (i.e. above −20 mV) the relative effects of the serum were far less (and the changes did not reach significance) because L-type Ca^2+^ currents are the dominant currents at these voltages [69].

## Discussion

Anti-Ro/SSA and anti-La/SSB autoantibodies have been associated with CHB for decades, but the precise targets for these autoantibodies remain incompletely understood. For example, maternal sera from CHB pregnancies were reported to affect both L- and T-type currents (*I*_Ca,T_) [47]. Arguments can be made for the involvement of both of these Ca^2+^ currents in CHB. Indeed, mice lacking α_1D_ (Cav1.3^−/−^) or α_1G_ (Cav3.1^−/−^) display heart block [53], [70] as well as altered Wenckebach cycle lengths (WBCL). On the other hand, mice lacking the α_1H_ T-type calcium channel (Cav3.2^−/−^) have shown no ECG alterations [71]. These latter findings led us to hypothesize that the α_1G_ T-type calcium channel might be a target of CHB autoantibodies.

Consistent with our hypothesis, IHC staining demonstrated that α_1G_ is expressed in the AV node and AV bundle of the fetal human heart (Figure 2). In addition, our immunofluorescence and confocal results demonstrate that α_1G_ is expressed on the surface of ventricular cardiomyocytes (Figure 4). Protein (Western Blotting and IPs) and mRNA expression results (Figure 3) further support the presence of α_1G_ in the fetal myocardium (AVJ, ventricle). However, the expression of α_1G_ in the fetal heart does not establish a functional role. In this regard, previous studies in rabbit sinoatrial cells have established that T-type Ca^2+^ current is blocked by Mibefradil, a T-type calcium channel blocker at low concentrations [72], [73]. Consistent with these functional effects on T-type current, we found that Mibefradil strongly affected the WBCL in newborn, but not adult, rabbit hearts. On the other hand, the T-type calcium channel blocker NiCl_2_, had no effect on WBCLs in newborn rabbit hearts at concentrations up to 300 µM. These pharmacological observations support the conclusion that the α_1G_ (Cav3.1)-dependent T-type Ca^2+^ current, rather than the α_1H_-dependent T-type Ca^2+^ current, is the dominant current in the AV node in newborns since the Ni^+^ sensitivity of α_1H_ channels (IC_50_ = 13 µM) is much lower than α_1G_ channels (IC_50_ = 250 µM). This contention is consistent with results showing that mice lacking α_1G_ channels (Cav3.1^−/−^) have decreased T-type currents (*I*_Ca,T_) in both sinoatrial (SAN) and AV nodal (AVN) cardiomyocytes, as well as slowed AV conduction [53], while mice lacking α_1H_ channels (Cav3.2^−/−^) show no such abnormalities [67]. Further, the differential WBCL response in adult and newborn rabbits matches observations in mouse models showing that α_1G_ and α_1H_ are differentially regulated during development, with expression reductions occurring with aging [74], a trend that we observe in our quantitative PCR studies (decrease in α_1G_ and slight decrease in α_1H_, Figure 3A). However, reports differ on developmental expression of α_1G_ and α_1H_ [75]. Human studies have demonstrated expression of both α_1G_ and α_1H_ channel transcripts in adult human heart [51], [76], and α_1G_ protein in adult heart sinus node [77], but ours is the first report of α_1G_ protein expression in the AV node of human fetal hearts. A limitation of our mRNA measurements, however, was sample numbers of human fetal hearts (one per time point). Clearly, further investigation of the temporal expression of α_1G_ in human fetal hearts will be necessary in order to understand developmental expression. We nevertheless demonstrate expression of α_1G_ protein in the AV node of 21 week-old fetal hearts, indicating that α_1G_ is present during the time period when CHB usually occurs in the fetus.

Involvement of α_1G_ in CHB is supported by a number of our present observations. Firstly, CHB^+^ sera recognized α_1G_ protein from human fetal hearts in Western blot. Further, the reactivity of CHB^+^ sera was epitope mapped to the α_1G_ p305 peptide (aa305–319), where we have identified an essential “core” signature sequence. However, although our ELISA screen identified clear patterns of reactivity/binding consistent with the need for a core sequence of 10 amino acids for recognition, a significant difference in reactivity of CHB^+^ vs. CHB^−^ sera was observed only for the 305/310 peptide, with considerable overlap between the two sera types. These observations suggest that maternal antibody reactivity to an additional target(s) may be involved in the disease.

Additional evidence for functional involvement of T-type currents in CHB was obtained by applying our CHB^+^ sera to adult mouse SAN cardiomyocytes, cells previously demonstrated to exhibit relatively high, and therefore easily measured, T-type Ca^2+^ currents [78]. Moreover, the T-type Ca^2+^ current in the adult mouse heart is primarily comprised of α_1G_-dependent channels [53]. Consistent with these previous studies, we observed expression of α_1G_ (Cav3.1) in the neonatal and adult mouse heart. CHB^+^ sera also recognized a protein with the same molecular weight in neonates and in adults, and was able to preferentially block Ca^2+^ currents activating at more negative voltages (from −60 to −30 mV) – known to correspond with activation of T-type channels at a more negative voltage [78]. Additionally, this block by CHB^+^ sera was not reversed on washout, consistent with high affinity binding, and no block was seen when CHB^−^ sera was added.

While collectively our results firmly support the conclusion that α_1G_ channels play a role in CHB in newborns, we cannot rule out the possible involvement of other channels. Indeed, maternal sera from CHB pregnancies have been shown to effect both L-type Ca^2+^ currents and α_1H_-based T-type Ca^2+^ currents [47]. Our peptide mapping studies of α_1G_ identified a short sequence located in the S5–S6 extracellular (P-loop) segment in repeat I as a likely potential antigenic epitope for CHB^+^ sera. Sequence alignment revealed that the other cardiac T-type calcium channel subunit, α_1H_, has a sequence with high similarity with the α_1G_ channel protein in this region, which also was recognized by CHB^+^ sera. Potential involvement of α_1H_-dependent T-type calcium channels in CHB is further supported by our observation that α_1H_ mRNA is expressed in fetal human hearts. Surface expression of α_1H_ in human fetal cardiomyocytes however, has not been demonstrated in this study. Accessibility of the α_1G_ extracellular epitope on ventricular cardiomyocytes was demonstrated by CHB maternal sera antibodies affinity-purified towards the p305 α_1G_ peptide surface staining non-permeabilized ventricular cardiomyocytes (Figure 4). After permeabilization, and staining for α_1G_, co-staining could be seen with a portion of α_1G_ present on the surface. Further studies are needed to determine the accessibility of the α_1H_ epitope for antibody binding in human fetal hearts and its functional significance. CHB^+^ sera has also been shown previously to interact with a recombinant protein derived from the S5–S6 extracellular region in repeat I of the L-type calcium channel alpha subunit, α_1D_ [48]. Although sequence alignments did not reveal a similar “core sequence”, it seems possible that the S5–S6 extracellular loop region in repeat I of both T-type and L-type calcium channels may have a conserved structure or, alternatively, this region may have a strong propensity for promoting immune responses, consistent with identification of this region of α_1G_ protein by ElliPro Epitope Prediction and the Kolaskar & Tongaonker Antigenicity as having a high likelihood of antigenicity. The possibility of multiple protein targeting in CHB requires further investigation, and characterization of sera antibody specificity with respect to anti-Ro52, anti-Ro60 and anti-La will be necessary to determine the fine specificity of the antibodies cross-reacting with these channels. In this regard, we wish to note that expansion beyond CHB of the clinical spectrum for mothers with anti-Ro/SSA and anti-La/SSB antibodies [3]–[9], [61] might be tied to the existence of a common epitope on several protein targets.

In summary, the data presented here implicate α_1G_ as one specific target of CHB maternal sera antibodies, and suggest the potential for involvement of α_1H_, although we note that further functional studies with human cardiomyocytes are necessary to determine this conclusively. Investigations into specific channel isoforms and modifications, that may impact antibody binding to the native human proteins, will be important in understanding which targets bound by antibodies are important for development of the human disease.

## Materials and Methods

### Patients

The study included sera from 28 women who were followed at the Hospital for Sick Children, Toronto, Canada. Seventeen of the samples were from mothers with anti-Ro/La autoantibodies and CHB affected pregnancies with AVB III (CHB^+^). Ten samples were from mothers with anti-Ro/La antibodies who were at risk for CHB, and seen at the rheumatology clinic, but with normal fetal heart rate (CHB^−^) outcome, evaluated by fetal echocardiography (ECG) during pregnancy, and 10 anti-Ro/La negative samples were used as healthy controls (HC). The Hospital for Sick Children Research Institute Research Ethics Board (REB) approved the study under protocol 0019970143 and written consent was obtained from mothers for use of the sera.

### Human fetal heart tissue

Human fetal hearts (17- to 23-week gestation) were obtained through the Heart Centre Biobank Program from elective terminations of normal pregnancies. The use of human fetal tissue was approved by the Hospital for Sick Children Research Institute REB under protocol 1000029263. Written consent was obtained in the clinic, and portions of fetal hearts from fetal terminations are sent under a material transfer agreement to undergo banking within the Heart Centre Biobank Registry program under REB protocol number 1000011232.

Fetal hearts were dissected by an experienced cardiologist following an established method [59]. In brief, hearts are immobilized in a dissecting dish, right ventricle uppermost, which is slit open to visualize the tricuspid inlet below the interatrial septum. The AV junction is identified using anatomical landmarks of the septal cusp of the tricuspid valve inferiorly, the coronary sinus posteriorly, the tendon of Todaro superiorly. Tissue was either dissociated for immunofluoresence, flash frozen for immunoprecipitation experiments, or quickly submerged in RNA stabilizing reagents for immediate RNA preparation.

### Preparation and perfusion of the Langendorff rabbit CHB model

The Animal Care Committee at the Hospital for Sick Children has approved the use of neonatal and adult rabbits as well as the experimental design of this study under protocol number 3250. The animals were housed in the Animal Care Facility at the Hospital for Sick Children and maintained in accordance with hospital regulations and CCAC guidelines. Rabbit hearts were prepared for perfusion as previously described [57]. Briefly, following loss of the pain reflex, the rabbit sternum was incised and removed. The beating heart was dissected, and perfused with fresh oxygenated modified Krebs-Henseleit solution. The flow rate was constant at 9 ml/min for neonates and 36 ml/min for adults, the temperature was kept at 37°C and the left ventricle was left unloaded. The sinus node at the level of the superior vena cava-right atrial junction was excised until junctional rhythm was obtained, following which the hearts were allowed to equilibrate for 15 min.

### Electrophysiologic recording for WBCL analysis

In order to compare calcium channel blocker effects on newborn versus mature AV nodal conduction, the rabbit Langendorff heart model was modified by atrial pacing the preparation at sequentially shorter cycle lengths until the AV nodal Wenckebach cycle length (WBCL) was identified, as described previously [57]. At baseline and 15 minutes following equilibration of each concentration of channel blocker, the atrium was paced at incrementally faster rates to determine WBCL. Finally, the hearts were perfused with fresh modified Krebs-Henseleit solution for 15 min and paced to determine WBCL in the washout state. The WBCL was defined as the slowest atrial paced rate with any failure of AV conduction within 8 consecutive seconds of pacing.

### Calcium channel blockers

Prior to the comparative study, we assessed the effect of each blocker on WBCL prolongation in neonatal rabbit Langendorff preparations. We chose the concentration of each drug to cause at least double prolongation of WBCL in newborn rabbits. Adults (n = 7) and newborn (n = 8) hearts were exposed to two concentrations of either I_f_ blocker (ZD7288; 10, 20 µM), I_Ca-L_ blockers (Verapamil; 0.2, 0.4 µM. Diltiazem; 0.4, 0.8 µM) or I_Ca-T_ blocker (Mibefradil; 0.4,0.8 µM). Increasing concentrations of a single drug were added to the perfusate in a sequential cumulative pattern. Newborn hearts were also assessed for WBCL prolongation upon exposure to a specific blocker of the T-type calcium channel α_1H_ subunit (Nickel Chloride; 1, 10, 100 and 300 µM).

### Histology and Immunohistochemistry

AVJ was dissected and fixed in 4% paraformaldehyde. After fixation, AVJ tissue was preserved as paraffin-embedded blocks. Slides were made from the blocks and underwent Masson's trichrome stain and were immunolabelled for α_1G_ and NF-160. The slides were dewaxed, rehydrated and rinsed 2×5 min in double distilled water. The slides were then incubated with Target Retrieval Solution (DAKO Canada) at 95°C for 40 min and then room temperature 20 min. After rinsing 2× with PBS, sections were blocked with blocking buffer (PBS with 3% fish gelatin, 2 mg/ml BSA and 2% Tween-20) for one hour, and incubated with rabbit-anti-α_1G_ (Novus Biologicals Canada) at a dilution of 1∶400 and mouse anti-NF-160 (1∶500, US Biological) for 2 hours. After rinsing 3× with PBS, slides were then incubated in the dark with donkey-anti-rabbit Cy3 (1∶1000, Jackson Immunolabs) and donkey anti-mouse Alexa488 (1∶1000, Invitrogen) for 40 min, DAPI was then added in the last 5 min, slides were washed 3×5 min in PBS and mounted with a coverglass. Slides were examined using a Zeiss Epifluorescence microscope.

### Real time PCR

Total RNA was prepared according to the protocol *RNeasy Fibrous Tissue Mini Kit* (Qiagen). Homogenization was performed with a Tissue Tearor. RNA concentration and purity was determined and purified total RNA was converted to cDNA with Superscript III reverse transcriptase (Invitrogen). Power SYBR Green PCR Master Mix (Applied Biosystems) was used to perform relative quantification of target sequences *CACNA1G* (forward primer: CAAACTTGTGGCCTTTGGTT, reverse primer: GGTGGACTCCTGGTCACAGT) and *CACNA1H* (forward primer: ATAACCAACCCAAGTCGCTG, reverse primer: CAGGAGCATGAAAAGAAGGC) compared to the housekeeping gene *GAPDH* (forward primer: CCTGTTCGACAGTCAGCCGCATC, reverse primer: GGTGACCAGGCGCCCAATACG). Assay was optimized for the cDNA amount as well as PCR conditions to validate the primers and ascertain equal efficiency of primers. Samples were prepared in triplicates and subjected to default conditions for the 7000 Sequence Detection System by ABI (annealing at 60°C for 1 minute).

### Immunoprecipitation, Western blot

Fresh tissue from 20–22 week-old fetal human hearts were homogenized in NP40 lysis buffer (1% NP40, 0.15M NaCl, 0.01M Na_3_PO_4_ pH 7.2, 2.5 mM Na_4_O_7_P_2_, 2 mM EDTA, 1 mM Na_3_VO_4_, 1 mM PMSF) with proteinase inhibitor cocktail (Roche, Penzberg, Germany). Homogenized lysates were prepared at room temperature, incubated for 30 min and then centrifuged at 12,000 rpm for 15 min. Protein concentration was determined with the Bradford protein assay (Bio-Rad, Hercules, CA, USA) and 1 mg of total protein lysates were used per immunoprecipitation sample. Immunoprecipitation of α_1G_ protein was achieved with Protein G Sepharose beads and 2 µg of α_1G_ antibody (Cav3.1 antibody, Alamone Labs). Samples were incubated at 70°C for 5 min in Laemmli loading buffer. Samples were loaded onto 10% Tris-glycine sodium dodecyl sulfate polyacrylamide gels electrophoresed at 120 V for 2.5 h, and transferred overnight at room temperature (20 V) to nitrocellulose membranes. Membranes were blocked with 5% milk in Tris-buffered saline/0.05% Tween-20 (TBS-T), then probed with either human sera (1∶100), anti-Cav3.1 antibody (1∶200 dilution, Santa Cruz for Western blot probing after IP, 1∶100 dilution, Sigma for western probing), or anti-Cav3.2 antibody (1∶200 dilution, Sigma) for 2 h at room temperature. For pre-incubation to demonstrate specificity of commercial antibody for α_1G_, a peptide corresponding to aa1–22 of rat α_1G_ was added at the recommended concentration of 1∶1 with the antibody prior to addition to the blot. Membranes were washed between each step 3×15 min with TBS-T. Membranes were incubated 1 h with anti-human IgG-HRP (1∶10,000 dilution, Jackson Immunolabs) or goat anti-rabbit IgG-HRP (1∶5,000 dilution Jackson Immunolabs). Anti-calnexin (CNX) antibody was used as loading control for Western blot. Membranes were developed with ECL substrate (Santa Cruz Biotechnology) and film exposed for visualization of bands. Anti-Ro and anti-La titers given for maternal sera used in immunoassays are from routine clinical screening based on an ELISA using recombinant human anti-Ro60-kD and anti-Ro52-kD, and anti-La 48-kD protein (Phadia GmbH, Freiburg, Germany).

### Immunofluorescence

Dissected regions of fetal heart tissue was washed in Hank's solution (HBSS) and incubated with gentle shaking overnight in Hank's solution supplemented with 5 mM BDM and 1 mg/ml collagenase. Heart pieces were minced and incubated in fresh mincing solution (HBSS with 10 mM taurine, 0.1 mM EGTA, 10 mM BDM, 1 mg/ml BSA, 1 mg/ml collagenase) gently spinning with a small magnet at 37°C to harvest cells for 5 min. This procedure was repeated 3 times. Cells were washed then fixed with 2% paraformaldehyde for 10 min, shaking slowly for subsequent immunofluoresence labeling.

For detection of α_1G_ T-type calcium channel, rabbit anti-human Cav3.1 antibody was used at a dilution of 1∶500 (Novus Biologicals Canada), and for detection of cardiomyocyte markers mouse anti-human cardiac Troponin-T was used at a dilution of 1∶500 (Thermo Scientific). Cells were resuspended in blocking buffer (PBS with 3% fish gelatin, 2 mg/ml BSA, 0.05% Tween-20) at room temperature for 1 h. Primary antibodies were added overnight at 4°C, shaking gently. After 2×15 min washes in PBS, cells were incubated in the dark for 4 h at 4°C with secondary antibodies: donkey anti-rabbit-IgG-Cy3 (1∶5,000 Jackson ImmunoResearch) and donkey anti-mouse-IgG-Alexa488 (1∶1,000 Green Invitrogen). DAPI stain was used to visualize nucleus (0.1 µg/ml). Cells were washed 2×15 min in PBS and mounted on glass for confocal microscopy (60× magnification).

For the surface staining with affinity-purified sera, non-permeabilized cells were blocked (PBS with 3% fish gelatin, 2 mg/ml BSA) at room temperature for 1 h before incubation with Cav3.1 antibody. Affinity purified sera was diluted in blocking buffer (without detergent) 1∶2 (as already diluted from elution/neutralization buffers) for 2 h at room temperature. Cardiomyocytes were washed and secondary antibody (rabbit anti-human-IgG-Cy3 at 1∶1,000, Jackson Immunolabs) was added, and cells were incubated for 1 h at room temperature in the dark. Subsequent staining for the α_1G_ T-type calcium channel followed the procedure described above, except that the secondary antibody used was donkey anti-rabbit-IgG-Dylight488 (1∶1,000 Jackson ImmunoResearch).

### Affinity Purification of Antibodies from Maternal Sera Specific for the p305 Peptide

Serum was affinity purified towards the α_1G_ p305 peptide (aa305–319 of α_1G_) for use in immunofluorescence experiments as follows: A streptavidin resin filled column was equilibrated and washed with PBS. Serum was pre-incubated overnight with biotinylated p305 peptide. Free biotin was then added to the serum solution to block all non-specific biotin binding sites, after which the solution was applied to the streptavidin column. The column was then washed 3 times with PBS to remove antibodies not specific for peptide, and antibodies of interest were then eluted with 0.1 M glycine, pH 2.5 and immediately transferred into 1M Tris, pH 8.0 in a 1∶10 ratio.

### Peptides and ELISA

ELISA epitope mapping was performed using α_1G_ peptide PEPscreen Custom peptide libraries (Sigma, St. Louis, MO, USA). We investigated fifty overlapping 15aa peptides corresponding to aa 130–385. Thirty-six 15aa peptides corresponding to aa774–963, and forty-four 15aa peptides covering aa1308–1536 were studied. Peptides were dissolved (80% DMSO/20% water) and 96-well ELISA plates were coated with antigen (20 µg/ml) in carbonate coating buffer pH 9.6 (0.03 M Na_2_CO_3_, 0.07 M NaHCO_3_, 0.1% NaN_3_), and incubated overnight at 4°C. Plates were washed and blocked with 5% BSA for 2 h and incubated for 2 h with sera at room temperature. Plates were washed 3× with washing buffer and incubated for 1 h with goat anti-human IgG AP-conjugate (Sigma, St. Louis, MO, USA). Plates were then developed with AP substrate buffer (R&D systems) and read at OD 405 nm minus that of the reagent blank. All samples were run in duplicate. Controls were from 10 anti-Ro/La positive mothers who were at-risk for CHB and followed in the rheumatology clinic, but with healthy pregnancy outcomes, as well as control sera (anti-Ro/La negative) for assay development. Cut-offs for epitope mapping were determined by calculation of HC average+3× standard deviation.

The time at which ELISA experiments were read by spectrophotometer OD (405 nm) was optimized for each peptide using two criteria: (i) OD_405_<0.3 in healthy control samples, and (ii) OD_405_ as high as possible among test samples within spectrophotometric limits (OD_405_<2.0). OD values shown in Figures 7 and 8 were accordingly obtained at various incubation times. Peptide ELISA reading times were as follows: 50 min for p305; 2 h 30 min for p300 and p315; 1 hr 30 min for p310 and p305/p310.

Comparison of α_1G_ and α_1H_ peptides was accomplished with peptide amines modified at their N- and C-termini with lysine residues to enhance water solubility [79] (α_1G_, sequence H_2_N-KKKYNSSSNTTCVNWNQYKKK-NH_2_; α_1H_, H_2_N-KKKVGAARNACINWNQYKKK-NH_2_). These peptides were dissolved in water and coated at 1 µg/ml and blocked with 1% fish serum for 2 h before incubating with primary sera for 2 h. Otherwise the procedure was identical to the above.

### Electrophysiological recordings of T-type calcium current

Mice were cared for and housed in the Animal Care Facility at University of Toronto and maintained in accordance with university regulations and Canadian Council on Animal Care (CCAC) guidelines. The use of mice for electrophysiology studies was approved by The Animal Care Committee of University of Toronto under Protocol 20009885.

After heparinized mouse (C57BL/6, 8 weeks old, male, Charles River Inc. Canada) was anesthetized, heart was quickly removed and retrogradely perfused through aorta with 37°C Ca^2+^-free Tyrode's solution, containing (in mM) 137 NaCl, 5.4 KCl, 1.0 MgCl_2_, 0.33 NaH_2_PO_4_, 22 D-glucose, 10 HEPES, pH 7.3, for 8∼10 min. Then, collagenase (1.0 mg/mL, Worthington, type II) and elastase (0.15 mg/mL, Worthington) were added. Heart was digested for 35∼50 min. SAN region (as described in [80]) was cut and cells were dissociated and kept in solution which contained (in mM) 120 potassium glutamate, 20 KCl, 20 HEPES, 1.0 MgCl_2_, 10 D-glucose, 0.5 K-EGTA, and 0.1% bovine serum albumin, pH 7.35 with KOH.

Ca^2+^ current was introduced by 400 ms test pulse from −70 mv to +30 mV with 10 mV step from holding potential at −80 mV or −40 mV. Cells were bathed in solution containing (in mM) 140 CsCl, 1 MgCl_2_, 1.8 CaCl_2_ 10 HEPES, 10 D-glucose, pH 7.35 with CsOH. SAN cells were picked as described previously [68]. Whole-cell patch clamp recording (Axopatch 200B and Clampex 8 software, Axon Instrument, CA, USA) was performed with 3.0∼4 MΩ pipette when filled with solution containing (in mM): 135 CsCl, 5 TEA, 1 MgCl_2_, 4 MgATP, 10 HEPES, 10 EGTA, 0.3 Na_2_GTP, pH 7.2 with CsOH. Serial resistance was 80%∼85% compensated. Data were analyzed with Clampfit 10 (Axon Instrument, CA, USA).

In order to evaluate the effect of serum on T-type calcium current, positive (CHB^+^) or control serum (normal serum) was added into external solution (1∶50 dilution) and kept for 5 min for full reaction, then serum was fully washed out to remove Na^+^ from external solution.

### Statistical analysis

For ELISA assay, Mann-Whitney U-test or Kruskall-Wallis ANOVA were used for statistical analysis. Mann-Whitney analysis was used for gene expression comparison. For analysis of electrophysiology data a paired T-test was used. The level of significance was set at p<0.05.

## References

[1] Buyon JP, Hiebert R, Copel J, Craft J, Friedman D, et al (1998) Autoimmune-associated congenital heart block: demographics, mortality, morbidity and recurrence rates obtained from a national neonatal lupus registry. Journal of the American College of Cardiology 31: 1658–1666. 9626848 10.1016/s0735-1097(98)00161-2

[2] Friedman DM, Rupel A, Buyon JP (2007) Epidemiology, etiology, detection, and treatment of autoantibody-associated congenital heart block in neonatal lupus. Curr Rheumatol Rep 9: 101–108. 17502039 10.1007/s11926-007-0003-4

[3] Chockalingam P, Jaeggi ET, Rammeloo LA, Haak MC, Adama van Scheltema PN, et al (2011) Persistent fetal sinus bradycardia associated with maternal anti-SSA/Ro and anti-SSB/La antibodies. J Rheumatol 38: 2682–2685. 22089457 10.3899/jrheum.110720

[4] Jaeggi ET, Hamilton RM, Silverman ED, Zamora SA, Hornberger LK (2002) Outcome of children with fetal, neonatal or childhood diagnosis of isolated congenital atrioventricular block. A single institution's experience of 30 years. J Am Coll Cardiol 39: 130–137. 11755298 10.1016/s0735-1097(01)01697-7

[5] Eronen M, Heikkila P, Teramo K (2001) Congenital complete heart block in the fetus: hemodynamic features, antenatal treatment, and outcome in six cases. Pediatr Cardiol 22: 385–392. 11526412 10.1007/s002460010256

[6] Taylor-Albert E, Reichlin M, Toews WH, Overholt ED, Lee LA (1997) Delayed dilated cardiomyopathy as a manifestation of neonatal lupus: case reports, autoantibody analysis, and management. Pediatrics 99: 733–735. 9113953 10.1542/peds.99.5.733

[7] Nield LE, Silverman ED, Taylor GP, Smallhorn JF, Mullen JB, et al (2002) Maternal anti-Ro and anti-La antibody-associated endocardial fibroelastosis. Circulation 105: 843–848. 11854125 10.1161/hc0702.104182

[8] Nield LE, Silverman ED, Smallhorn JF, Taylor GP, Mullen JB, et al (2002) Endocardial fibroelastosis associated with maternal anti-Ro and anti-La antibodies in the absence of atrioventricular block. J Am Coll Cardiol 40: 796–802. 12204513 10.1016/s0735-1097(02)02004-1

[9] Lazzerini PE, Capecchi PL, Acampa M, Morozzi G, Bellisai F, et al (2011) Anti-Ro/SSA-associated QTc interval prolongation in the adults: The role of antibody level and Specificity. Arthritis Care Res (Hoboken). 10.1002/acr.20540 21739618

[10] Buyon JP, Hiebert R, Copel J, Craft J, Friedman D, et al (1998) Autoimmune-associated congenital heart block: demographics, mortality, morbidity and recurrence rates obtained from a national neonatal lupus registry. J Am Coll Cardiol 31: 1658–1666. 9626848 10.1016/s0735-1097(98)00161-2

[11] Finkelstein Y, Adler Y, Harel L, Nussinovitch M, Youinou P (1997) Anti-Ro (SSA) and anti-La (SSB) antibodies and complete congenital heart block. 148: 205–208. 9255327

[12] Groves AM, Allan LD, Rosenthal E (1996) Outcome of isolated congenital complete heart block diagnosed in utero. Heart 75: 190–194. 8673760 10.1136/hrt.75.2.190 PMC484258

[13] Machado MV, Tynan MJ, Curry PV, Allan LD (1988) Fetal complete heart block. British Heart Journal 60: 512–515. 3224055 10.1136/hrt.60.6.512 PMC1224893

[14] Schmidt KG, Ulmer HE, Silverman NH, Kleinman CS, Copel JA (1991) Perinatal outcome of fetal complete atrioventricular block: a multicenter experience. J Am Coll Cardiol 17: 1360–1366. 2016455 10.1016/s0735-1097(10)80148-2

[15] Lopes LM, Tavares GM, Damiano AP, Lopes MA, Aiello VD, et al (2008) Perinatal outcome of fetal atrioventricular block: one-hundred-sixteen cases from a single institution. Circulation 118: 1268–1275. 18765396 10.1161/CIRCULATIONAHA.107.735118

[16] Jaeggi ET, Golding F, Silverman ED (2009) Letter by Jaeggi et al regarding article, “Perinatal outcome of fetal atrioventricular block: one-hundred sixteen cases from a single institution”. Circulation 119: e539 author reply e541–532. 19470895 10.1161/CIRCULATIONAHA.108.830877

[17] Brucato A, Frassi M, Franceschini F, Cimaz R, Faden D, et al (2001) Risk of congenital complete heart block in newborns of mothers with anti-Ro/SSA antibodies detected by counterimmunoelectrophoresis: a prospective study of 100 women. Arthritis and Rheumatism 44: 1832–1835. 11508435 10.1002/1529-0131(200108)44:8<1832::AID-ART320>3.0.CO;2-C

[18] Eronen M, Siren MK, Ekblad H, Tikanoja T, Julkunen H, et al (2000) Short- and long-term outcome of children with congenital complete heart block diagnosed in utero or as a newborn. Pediatrics 106: 86–91. 10878154 10.1542/peds.106.1.86

[19] Brucato A, Franceschini F, Gasparini M, De Juli E, Ferraro G, et al (1995) Isolated congenital complete heart block: longterm outcome of mothers, maternal antibody specificity and immunogenetic background. J Rheumatol 22: 533–540. 7783076

[20] Strandberg L, Salomonsson S, Bremme K, Sonesson S, Wahren-Herlenius M (2006) Ro52, Ro60 and La IgG autoantibody levels and Ro52 IgG subclass profiles longitudinally throughout pregnancy in congenital heart block risk pregnancies. Lupus 15: 346–353. 16830880 10.1191/0961203306lu2309oa

[21] Buyon JP (1993) Congenital complete heart block. Lupus 2: 291–295. 8305921 10.1177/096120339300200503

[22] Julkunen H, Kurki P, Kaaja R, Heikkila R, Immonen I, et al (1993) Isolated congenital heart block - Long-term outcome of mothers and characterization of the immune response to SS-A/Ro and to SS-B/La. Arthritis Rheum 36: 1588–1598. 8240435 10.1002/art.1780361114

[23] Brucato A, Gasparini M, Vignati G, Riccobono S, De Juli E, et al (1995) Isolated congenital complete heart block: longterm outcome of children and immunogenetic study. The Journal of Rheumatology 22: 541–543. 7783077

[24] Dorner T, Chaoui R, Feist E, Goldner B, Yamamoto K, et al (1995) Significantly increased maternal and fetal IgG autoantibody levels to 52 kD Ro (SS-A) and La(SS-B) in complete congenital heart block. Journal of Autoimmunity 8: 675–684. 8579723 10.1006/jaut.1995.0050

[25] Silverman ED, Buyon J, Laxer RM, Hamilton R, Bini P, et al (1995) Autoantibody response to the Ro/La particle may predict outcome in neonatal lupus erythematosus. Clinical and Experimental Immunology 100: 499–505. 7774062 10.1111/j.1365-2249.1995.tb03729.x PMC1534456

[26] Julkunen H, Kaaja R, Siren MK, Mack C, McCready S, et al (1998) Immune-mediated congenital heart block (CHB): identifying and counseling patients at risk for having children with CHB. Semin Arthritis Rheum 28: 97–106. 9806370 10.1016/s0049-0172(98)80042-5

[27] Salomonsson S, Dorner T, Theander E, Bremme K, Larsson P, et al (2002) A serologic marker for fetal risk of congenital heart block. Arthritis and Rheumatism 46: 1233–1241. 12115229 10.1002/art.10232

[28] Brucato A, Frassi M, Franceschini F, Cimaz R, Faden D, et al (2001) Risk of congenital complete heart block in newborns of mothers with anti-Ro/SSA antibodies detected by counterimmunoelectrophoresis: a prospective study of 100 women. Arthritis Rheum 44: 1832–1835. 11508435 10.1002/1529-0131(200108)44:8<1832::AID-ART320>3.0.CO;2-C

[29] Salomonsson S, Sonesson SE, Ottosson L, Muhallab S, Olsson T, et al (2005) Ro/SSA autoantibodies directly bind cardiomyocytes, disturb calcium homeostasis, and mediate congenital heart block. J Exp Med 201: 11–17. 15630133 10.1084/jem.20041859 PMC2212767

[30] Strandberg L, Winqvist O, Sonesson SE, Mohseni S, Salomonsson S, et al (2008) Antibodies to amino acid 200–239 (p200) of Ro52 as serological markers for the risk of developing congenital heart block. Clin Exp Immunol 154: 30–37. 18727629 10.1111/j.1365-2249.2008.03732.x PMC2561087

[31] Jaeggi E, Laskin C, Hamilton R, Kingdom J, Silverman E (2010) The importance of the level of maternal anti-Ro/SSA antibodies as a prognostic marker of the development of cardiac neonatal lupus erythematosus a prospective study of 186 antibody-exposed fetuses and infants. J Am Coll Cardiol 55: 2778–2784. 20538173 10.1016/j.jacc.2010.02.042

[32] Silverman ED, Buyon J, Laxer RM, Hamilton R, Bini P, et al (1995) Autoantibody response to the Ro/La particle may predict outcome in neonatal lupus erythematosus. Clin Exp Immunol 100: 499–505. 7774062 10.1111/j.1365-2249.1995.tb03729.x PMC1534456

[33] Reed JH, Neufing PJ, Jackson MW, Clancy RM, Macardle PJ, et al (2007) Different temporal expression of immunodominant Ro60/60 kDa-SSA and La/SSB apotopes. Clin Exp Immunol 148: 153–160. 17286801 10.1111/j.1365-2249.2007.03331.x PMC1868853

[34] Litsey SE, Noonan JA, OConnor WN, Cottrill CM, Mitchell B (1985) Maternal connective tissue disease and congenital heart block. Demonstration of immunoglobulin in cardiac tissue. N-Engl-J-Med 312: P 98–100. 10.1056/NEJM198501103120206 3880599

[35] Ho SY, Esscher E, Anderson RH, Michaelsson M (1986) Anatomy of congenital complete heart block and relation to maternal anti-Ro antibodies. The American Journal of Cardiology 58: 291–294. 3090867 10.1016/0002-9149(86)90064-0

[36] Clancy RM, Kapur RP, Molad Y, Askanase AD, Buyon JP (2004) Immunohistologic evidence supports apoptosis, IgG deposition, and novel macrophage/fibroblast crosstalk in the pathologic cascade leading to congenital heart block. Arthritis Rheum 50: 173–182. 14730614 10.1002/art.11430

[37] Brucato A, Cimaz R, Catelli L, Meroni P (2000) Anti-Ro-associated sinus bradycardia in newborns. Circulation 102: E88–89. 10982556 10.1161/01.cir.102.11.e88

[38] Menon A, Silverman ED, Gow RM, Hamilton RM (1998) Chronotropic competence of the sinus node in congenital complete heart block. American Journal of Cardiology 82: 1119–1121, A1119. 9817493 10.1016/s0002-9149(98)00569-4

[39] Hu K, Qu Y, Yue Y, Boutjdir M (2004) Functional basis of sinus bradycardia in congenital heart block. Circ Res 94: e32–38. 14963005 10.1161/01.RES.0000121566.01778.06

[40] Mazel JA, El Sherif N, Buyon J, Boutjdir M (1999) Electrocardiographic abnormalities in a murine model injected with IgG from mothers of children with congenital heart block. Circulation 99: 1914–1918. 10199891 10.1161/01.cir.99.14.1914

[41] Boutjdir M, Chen L, Zhang ZH, Tseng CE, DiDonato F, et al (1997) Arrhythmogenicity of IgG and anti-52-kD SSA/Ro affinity-purified antibodies from mothers of children with congenital heart block. Circ Res 80: 354–362. 9048655 10.1161/01.res.80.3.354

[42] McCue CM, Mantakas ME, Tingelstad JB, Ruddy S (1977) Congenital heart block in newborns of mothers with connective tissue disease. Circulation 56: 82–90. 301070 10.1161/01.cir.56.1.82

[43] Eftekhari P, Salle L, Lezoualc'h F, Mialet J, Gastineau M, et al (2000) Anti-SSA/Ro52 autoantibodies blocking the cardiac 5-HT4 serotoninergic receptor could explain neonatal lupus congenital heart block. Eur J Immunol 30: 2782–2790. 11069058 10.1002/1521-4141(200010)30:10<2782::AID-IMMU2782>3.0.CO;2-9

[44] Eftekhari P, Roegel JC, Lezoualc'h F, Fischmeister R, Imbs JL, et al (2001) Induction of neonatal lupus in pups of mice immunized with synthetic peptides derived from amino acid sequences of the serotoninergic 5-HT4 receptor. Eur J Immunol 31: 573–579. 11180122 10.1002/1521-4141(200102)31:2<573::aid-immu573>3.0.co;2-9

[45] Qu Y, Baroudi G, Yue Y, Boutjdir M (2005) Novel molecular mechanism involving alpha1D (Cav1.3) L-type calcium channel in autoimmune-associated sinus bradycardia. Circulation 111: 3034–3041. 15939813 10.1161/CIRCULATIONAHA.104.517326

[46] Qu Y, Xiao GQ, Chen L, Boutjdir M (2001) Autoantibodies from mothers of children with congenital heart block downregulate cardiac L-type Ca channels. J Mol Cell Cardiol 33: 1153–1163. 11444920 10.1006/jmcc.2001.1379

[47] Xiao GQ, Hu K, Boutjdir M (2001) Direct inhibition of expressed cardiac l- and t-type calcium channels by igg from mothers whose children have congenital heart block. Circulation 103: 1599–1604. 11257091 10.1161/01.cir.103.11.1599

[48] Karnabi E, Qu Y, Wadgaonkar R, Mancarella S, Yue Y, et al (2010) Congenital heart block: identification of autoantibody binding site on the extracellular loop (domain I, S5–S6) of alpha(1D) L-type Ca channel. J Autoimmun 34: 80–86. 19640679 10.1016/j.jaut.2009.06.005 PMC2822065

[49] Karnabi E, Qu Y, Mancarella S, Boutjdir M (2011) Rescue and worsening of congenital heart block-associated electrocardiographic abnormalities in two transgenic mice. J Cardiovasc Electrophysiol 22: 922–930. 21352396 10.1111/j.1540-8167.2011.02032.x PMC3135711

[50] Mizuta E, Shirai M, Arakawa K, Hidaka K, Miake J, et al (2010) Different distribution of Cav3.2 and Cav3.1 transcripts encoding T-type Ca(2+) channels in the embryonic heart of mice. Biomed Res 31: 301–305. 21079360 10.2220/biomedres.31.301

[51] Monteil A, Chemin J, Bourinet E, Mennessier G, Lory P, et al (2000) Molecular and functional properties of the human alpha(1G) subunit that forms T-type calcium channels. J Biol Chem 275: 6090–6100. 10692398 10.1074/jbc.275.9.6090

[52] Greener ID, Monfredi O, Inada S, Chandler NJ, Tellez JO, et al (2011) Molecular architecture of the human specialised atrioventricular conduction axis. J Mol Cell Cardiol 50: 642–651. 21256850 10.1016/j.yjmcc.2010.12.017

[53] Mangoni ME, Traboulsie A, Leoni AL, Couette B, Marger L, et al (2006) Bradycardia and slowing of the atrioventricular conduction in mice lacking CaV3.1/alpha1G T-type calcium channels. Circ Res 98: 1422–1430. 16690884 10.1161/01.RES.0000225862.14314.49

[54] Suzuki H, Silverman ED, Wu X, Borges C, Zhao S, et al (2005) Effect of maternal autoantibodies on fetal cardiac conduction: an experimental murine model. Pediatr Res 57: 557–562. 15695601 10.1203/01.PDR.0000155947.82365.E4

[55] Strandberg LS, Ambrosi A, Jagodic M, Dzikaite V, Janson P, et al (2010) Maternal MHC Regulates Generation of Pathogenic Antibodies and Fetal MHC-Encoded Genes Determine Susceptibility in Congenital Heart Block. J Immunol 185: 3574–3582. 20696861 10.4049/jimmunol.1001396

[56] Ambrosi A, Dzikaite V, Park J, Strandberg L, Kuchroo VK, et al (2012) Anti-Ro52 monoclonal antibodies specific for amino acid 200–239, but not other Ro52 epitopes, induce congenital heart block in a rat model. Ann Rheum Dis 71: 448–454. 22084395 10.1136/annrheumdis-2011-200414

[57] Hamilton RM, Lee Poy M, Kruger K, Silverman ED (1998) Investigative methods of congenital complete heart block. Journal of Electrocardiology 30 Suppl: 69–74. 9535483 10.1016/s0022-0736(98)80035-6

[58] Lee JH, Gomora JC, Cribbs LL, Perez-Reyes E (1999) Nickel block of three cloned T-type calcium channels: low concentrations selectively block alpha1H. Biophys J 77: 3034–3042. 10585925 10.1016/S0006-3495(99)77134-1 PMC1300574

[59] Hancox JC, Levi AJ, Lee CO, Heap P (1993) A method for isolating rabbit atrioventricular node myocytes which retain normal morphology and function. The American Journal of Physiology 265: H755–766. 8368377 10.1152/ajpheart.1993.265.2.H755

[60] Rothenberg F, Efimov IR (2006) Three-dimensional anatomy of the conduction system of the early embryonic rabbit heart. Anat Rec A Discov Mol Cell Evol Biol 288: 3–7. 16287158 10.1002/ar.a.20244

[61] Jaeggi ET, Hornberger LK, Smallhorn JF, Fouron JC (2005) Prenatal diagnosis of complete atrioventricular block associated with structural heart disease: combined experience of two tertiary care centers and review of the literature. Ultrasound Obstet Gynecol 26: 16–21. 15937969 10.1002/uog.1919

[62] Swartz KJ (2008) Sensing voltage across lipid membranes. Nature 456: 891–897. 19092925 10.1038/nature07620 PMC2629456

[63] Karmazinova M, Beyl S, Stary-Weinzinger A, Suwattanasophon C, Klugbauer N, et al (2010) Cysteines in the loop between IS5 and the pore helix of Ca(V)3.1 are essential for channel gating. Pflugers Arch 460: 1015–1028. 20827487 10.1007/s00424-010-0874-5

[64] Ponomarenko J, Bui HH, Li W, Fusseder N, Bourne PE, et al (2008) ElliPro: a new structure-based tool for the prediction of antibody epitopes. BMC Bioinformatics 9: 514. 19055730 10.1186/1471-2105-9-514 PMC2607291

[65] Kolaskar AS, Tongaonkar PC (1990) A semi-empirical method for prediction of antigenic determinants on protein antigens. FEBS Lett 276: 172–174. 1702393 10.1016/0014-5793(90)80535-q

[66] Xiao GQ, Hu K, Boutjdir M (2001) Direct inhibition of expressed cardiac l- and t-type calcium channels by igg from mothers whose children have congenital heart block. Circulation 103: 1599–1604. 11257091 10.1161/01.cir.103.11.1599

[67] Hagiwara N, Irisawa H, Kameyama M (1988) Contribution of two types of calcium currents to the pacemaker potentials of rabbit sino-atrial node cells. J Physiol 395: 233–253. 2457676 10.1113/jphysiol.1988.sp016916 PMC1191991

[68] Mangoni ME, Nargeot J (2001) Properties of the hyperpolarization-activated current (I(f)) in isolated mouse sino-atrial cells. Cardiovasc Res 52: 51–64. 11557233 10.1016/s0008-6363(01)00370-4

[69] Catterall WA (2000) Structure and regulation of voltage-gated Ca2+ channels. Annu Rev Cell Dev Biol 16: 521–555. 11031246 10.1146/annurev.cellbio.16.1.521

[70] Zhang Z, He Y, Tuteja D, Xu D, Timofeyev V, et al (2005) Functional roles of Cav1.3(alpha1D) calcium channels in atria: insights gained from gene-targeted null mutant mice. Circulation 112: 1936–1944. 16172271 10.1161/CIRCULATIONAHA.105.540070

[71] Chen CC, Lamping KG, Nuno DW, Barresi R, Prouty SJ, et al (2003) Abnormal coronary function in mice deficient in alpha1H T-type Ca2+ channels. Science 302: 1416–1418. 14631046 10.1126/science.1089268

[72] Masumiya H, Kase J, Tanaka Y, Tanaka H, Shigenobu K (1999) Effects of mibefradil, a selective T-type Ca2+ channel antagonist, on sino-atrial node and ventricular myocardia. Res Commun Mol Pathol Pharmacol 104: 321–329. 10741382

[73] Mishra SK, Hermsmeyer K (1994) Selective inhibition of T-type Ca2+ channels by Ro 40–5967. Circ Res 75: 144–148. 8013072 10.1161/01.res.75.1.144

[74] Cribbs LL, Martin BL, Schroder EA, Keller BB, Delisle BP, et al (2001) Identification of the t-type calcium channel (Ca(v)3.1d) in developing mouse heart. Circ Res 88: 403–407. 11230107 10.1161/01.res.88.4.403

[75] Niwa N, Yasui K, Opthof T, Takemura H, Shimizu A, et al (2004) Cav3.2 subunit underlies the functional T-type Ca2+ channel in murine hearts during the embryonic period. Am J Physiol Heart Circ Physiol 286: H2257–2263. 14988077 10.1152/ajpheart.01043.2003

[76] Cribbs LL, Lee JH, Yang J, Satin J, Zhang Y, et al (1998) Cloning and characterization of alpha1H from human heart, a member of the T-type Ca2+ channel gene family. Circ Res 83: 103–109. 9670923 10.1161/01.res.83.1.103

[77] Chandler NJ, Greener ID, Tellez JO, Inada S, Musa H, et al (2009) Molecular architecture of the human sinus node: insights into the function of the cardiac pacemaker. Circulation 119: 1562–1575. 19289639 10.1161/CIRCULATIONAHA.108.804369

[78] Ono K, Iijima T (2010) Cardiac T-type Ca(2+) channels in the heart. J Mol Cell Cardiol 48: 65–70. 19729018 10.1016/j.yjmcc.2009.08.021

[79] Melnyk RA, Partridge AW, Yip J, Wu Y, Goto NK, et al (2003) Polar residue tagging of transmembrane peptides. Biopolymers 71: 675–685. 14991677 10.1002/bip.10595

[80] Mangoni ME, Couette B, Marger L, Bourinet E, Striessnig J, et al (2006) Voltage-dependent calcium channels and cardiac pacemaker activity: from ionic currents to genes. Prog Biophys Mol Biol 90: 38–63. 15979127 10.1016/j.pbiomolbio.2005.05.003

